# Highly Selective Methanol Synthesis Using Electrochemical CO_2_ Reduction with Defect‐Engineered Cu_58_ Nanoclusters

**DOI:** 10.1002/smsc.202400465

**Published:** 2024-11-28

**Authors:** Sourav Biswas, Tomoya Tanaka, Haohong Song, Masaki Ogami, Yamato Shingyouchi, Sakiat Hossian, Maho Kamiyama, Taiga Kosaka, Riki Nakatani, Yoshiki Niihori, Saikat Das, Tokuhisa Kawawaki, De‐en Jiang, Yuichi Negishi

**Affiliations:** ^1^ Research Institute for Science & Technology Tokyo University of Science 1‐3 Kagurazaka, Shinjuku‐ku Tokyo 162‐8601 Japan; ^2^ Department of Applied Chemistry Tokyo University of Science 1‐3 Kagurazaka, Shinjuku‐ku Tokyo 162‐8601 Japan; ^3^ Interdisciplinary Materials Science Vanderbilt University Nashville TN 37235 USA; ^4^ Carbon Value Research Center Tokyo University of Science 2641 Yamazaki, Noda Chiba 278‐8510 Japan; ^5^ Department of Chemical and Biomolecular Engineering Vanderbilt University Nashville TN 37235 USA; ^6^ Institute of Multidisciplinary Research for Advanced Materials Tohoku University Katahira 2‐1‐1, Aoba‐ku Sendai 980‐8577 Japan

**Keywords:** catalysis, copper, crystal structure, density functional theory, nanoclusters, thiolate

## Abstract

Atomically precise copper nanoclusters (Cu NCs) exhibit significant potential as catalysts for the electrocatalytic reduction of CO_2_. However, the range of products achievable with these NCs has been somewhat constrained. This study presents an innovative design strategy to enhance the catalytic activity of Cu NCs by engineering their active sites. These active sites are formed here by introducing defects on cubic Cu NCs through the partial dislocation of Cu atoms at their vertices, which creates surface ligand vacancies. This dislocation further refines the internal cationic geometry by altering cuprophilic interactions, leading to distinct modifications in the edges and vertices of the cubic geometry. These unique Cu(I) atom arrangements within the cluster effectively influence product specificity during electrochemical CO_2_ reduction. Density functional theory calculations correlate the enhanced selectivity for CH_3_OH in [Cu_58_H_20_(SPr)_36_(PPh_3_)_7_]^2+^ (Pr = CH_2_CH_2_CH_3_) NC to the increased reactivity of edge Cu atoms in binding CO and CHO intermediates, compared to [Cu_58_H_20_(SPr)_36_(PPh_3_)_8_]^2+^ and [Cu_58_H_20_(SEt)_36_(PPh_3_)_6_]^2+^ (Et = CH_2_CH_3_) NCs. Thus, this work underscores the potential of tailored structural designs of atomically precise nanocatalysts in directing electrochemical CO_2_ reduction toward unconventional products.

## Graphical abstract

This article explains how the structural architecture of nanomaterials influences product selectivity in the electrochemical CO_2_ reduction reaction. The study introduces an innovative design strategy to enhance the catalytic activity of Cu nanoclusters through active‐site engineering by generating defects in cubic Cu nanoclusters via partial dislocation of Cu atoms at their vertices, which creates surface ligand vacancies.

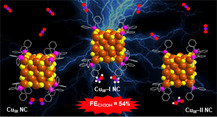

## 1 Introduction

The increasing emission of carbon dioxide (CO_2_) into the atmosphere is a major concern in the face of global warming and climate change.^[^ ^1^ ^]^ Despite efforts, the primary source—fossil fuel combustion to meet energy demands—continues unabated. This situation emphasizes the urgent need for innovative solutions for environmental degradation and energy sustainability.^[^ ^2^ ^]^ Amidst this challenge, a promising avenue emerges: harnessing the excess CO_2_ in our environment as an energy source.^[^ ^3^ ^]^ Among the diverse array of CO_2_‐reduction methodologies, the catalyst‐induced electrochemical reduction method has garnered significant attention.^[^ ^4^ ^]^ Once the reduction approach is established, the efficacy of the catalysts has become a paramount global research concern. Consequently, a burgeoning research endeavor is underway worldwide to engineer an optimal catalyst for this electrochemical reduction process.^[^ ^5^ ^]^ In the course of this journey, transition metal electrocatalysts have demonstrated their superiority over other contenders.^[^ ^6^ ^]^ However, unraveling the complexities of catalyst performance proves to be a formidable challenge, considering the influential role played by factors such as material structure and size.^[^ ^7^ ^]^ In response to this challenge, researchers are increasingly directing their attention toward achieving uniformity and smaller sizes, with nanoclusters (NCs) emerging as a promising avenue.^[^ ^8^ ^]^

Remarkably, gold and silver NCs have demonstrated notable success in efficiently reducing CO_2_ to CO, a valuable feedstock for the subsequent generation of fuel.^[^ ^9^ ^]^ However, the focus has shifted to the formation of other NCs for achieving products with even higher energy densities. Among the higher energy density products, methanol (CH_3_OH) emerges as a standout option, boasting exceptional characteristics such as high energy density, ease of storage, and transportability, making it highly desirable as a fuel source.^[^ ^10^ ^]^ Furthermore, the versatility of CH_3_OH extends beyond its role as a fuel. It serves as a raw material across a spectrum of industries including plastics, paints, silicone, and other chemical sectors, underscoring its pivotal importance. Despite its utility, the conventional steam methane reforming and dry reforming method of producing CH_3_OH emits substantial greenhouse gases under ultra‐high pressure and high‐temperature conditions, limiting its applicability in sustainable practices. Therefore, there is a growing interest in leveraging the electrochemical reduction of CO_2_ to selectively produce CH_3_OH under normal temperature and pressure, offering a near‐zero‐emission pathway toward achieving carbon neutrality.^[^ ^11^ ^]^

Recent research has shed light on the remarkable potential of copper (Cu) NCs in electrochemical CO_2_ reduction.^[^ ^12^ ^]^ Unlike traditional NC‐based catalysts, these Cu NCs possess a unique capability to yield predominant products such as formic acid (HCOOH), carbon monoxide (CO), and hydrocarbons during CO_2_ reduction.^[^ ^13^ ^]^ This breakthrough not only expands the repertoire of potential products but also signifies a substantial advancement in the pursuit of efficient CO_2_ reduction methodologies. While Cu nanoparticles have been utilized in this domain for their versatility in product generation, the utility of Cu NCs remains somewhat limited within certain parameters.^[^ ^14^ ^]^ Hence, there exists a compelling need to explore the potential of Cu NCs for producing a wider array of valuable products. It has been observed that various factors such as the NC architecture, number of constituent atoms, and ligands play pivotal roles in determining the catalytic activity of Cu NCs.^[^ ^12^, ^15^ ^]^ Additionally, defect sites on Cu NCs also play a crucial role in various catalytic reactions, although there is no study yet available on how these defects can tune the specificities of CO_2_‐reduction products.^[^ ^16^ ^]^ This presents an opportunity to fine‐tune the inherent properties of Cu NCs to produce additional beneficial compounds beyond the regular products efficiently.

In the context outlined earlier, we employed defect‐induced [Cu_58_H_20_(SPr)_36_(PPh_3_)_7_]^2+^ (Pr = CH_2_CH_2_CH_3_) (Cu_58_‐I) and [Cu_58_H_20_(SEt)_36_(PPh_3_)_6_]^2+^ (Et = CH_2_CH_3_) (Cu_58_‐II) NCs as catalysts for the highly selective electrochemical reduction of CO_2_. The remarkable selectivity of the reduction product and the catalytic activity primarily stem from the distinctive structural architecture of the catalyst. Comparative analysis with a regular [Cu_58_H_20_(SPr)_36_(PPh_3_)_8_]^2+^ (Cu_58_) NC highlights the superiority of the surface ligand vacancy and the consequent rearrangement of Cu(I) atoms. These structural features greatly enhance the efficacy of the electrochemical reduction approach for CO_2_. However, the specificities have some limitations, which are finely adjusted by the structural architecture of the NC catalyst. Theoretical studies have identified the active catalytic sites, which are closely associated with the vacant ligand sites. The rearrangement in these sites predominantly dictates the selectivity of the product, emphasizing the crucial role played by the architecture of the NC in governing the catalytic process.

## 2 Results and Discussion

### 2.1 Synthesis and Structural Architecture

Herein, we synthesized Cu_58_‐I and Cu_58_‐II NCs by following our previously reported protocol for Cu_58_ NC synthesis.^[^ ^17^ ^]^ In the case of Cu_58_‐I NC, the presence of one fewer PPh_3_ unit compared to the regular Cu_58_ NC is achieved by adjusting the composition of the ligands while maintaining the overall integrity of the NC architecture. However, when attempting to further decrease the number of PPh_3_ units to six, as seen in Cu_58_‐II NC, simply adjusting the ligand composition was insufficient. Instead, we achieved this by changing the thiolate ligand precursor from (SPr) to (SEt). The reduction in the carbon chain facilitated the stabilization of the NC despite the reduced number of PPh_3_ units.

In a typical synthesis, a Cu(I) precursor was subjected to treatment with PPh_3_ and specific thiol ligand in a solvent mixture containing acetonitrile and chloroform. The reaction proceeded until the addition of excess NaBH_4_. Upon completion, the resulting precipitate formed deep red‐colored block crystals through the slow evaporation of the solvent at room temperature. The single‐crystal X‐ray diffraction (SCXRD) revealed Cu_58_‐I NC crystallizes in a trigonal crystal system with a P‐3 (No. 147) space group (Table S1, Supporting Information). While Cu_58_‐II NC crystallizes in a triclinic crystal system with a P‐1 (No. 2) space group (Table S2, Supporting Information).

**Figure 1:**
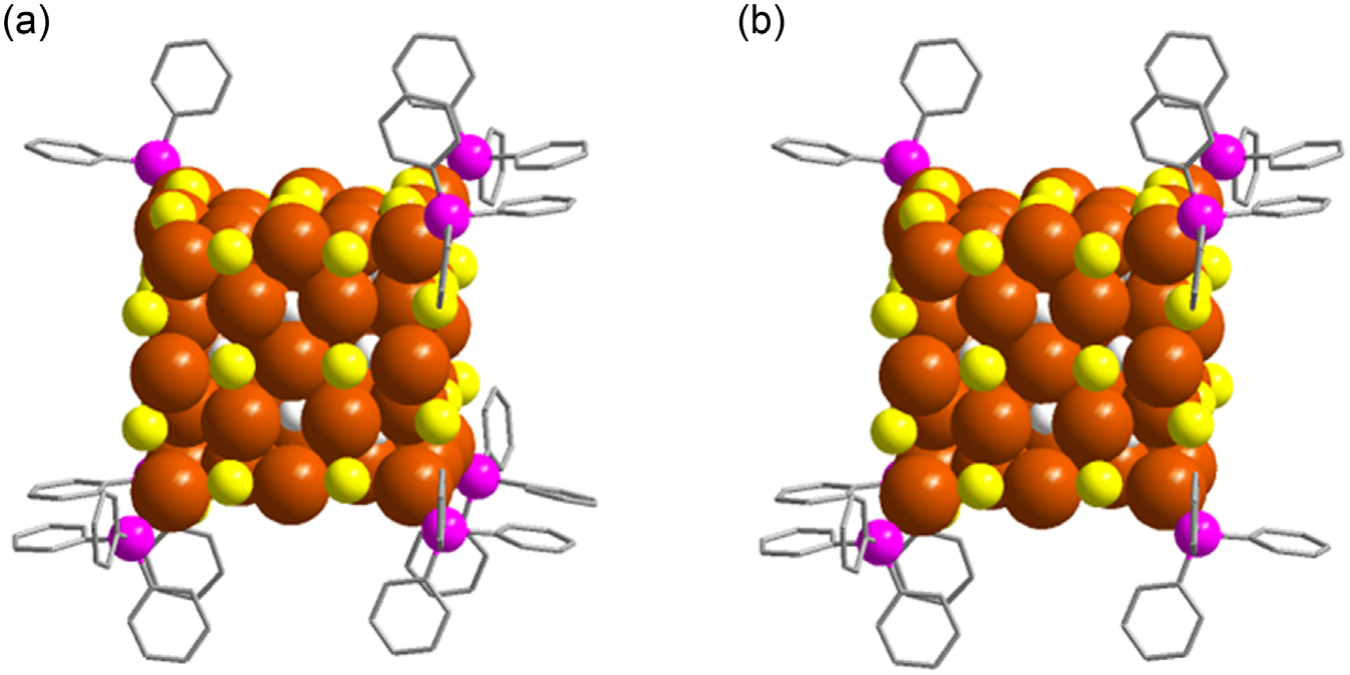
The overall structural architecture of a) Cu_58_‐I and b) Cu_58_‐II NCs. All the carbon parts are removed from the thiolate ligands, anionic part, and the solvent part also removed for the clarity. Hydrogen atoms are removed from the phenyl rings. Color legend: Cu, brown; S, yellow; P, violet; H, white; and C, grey stick.

**Figure 2:**
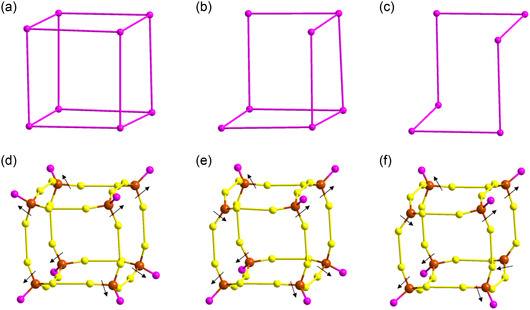
a) Complete cubic P_8_ shell of Cu_58_ NC, b) incomplete cubic P_7_ shell of Cu_58_‐I NC, c) incomplete cubic P_6_ shell of Cu_58_‐II NC, d) outward and inward directional arrangement of Cu_8_ cubic shell associated with the attached ligands in Cu_58_ NC, e) outward and inward directional arrangement of Cu_8_ cubic shell associated with the attached ligands in Cu_58_‐I NC and f) outward and inward directional arrangement of Cu_8_ cubic shell associated with the attached ligands in Cu_58_‐II NC. Bonds between the P—P and S—S are virtual. All the carbon parts are removed from the thiolate and PPh_3_ ligands. Color legend: Cu, brown; S, yellow; P, violet.

Although the obtained crystalline structures do not share a common crystal system, we observed a quite similar cubic arrangement of Cu(I) atoms which is surrounded by protective ligands in both Cu_58_‐I and Cu_58_‐II NCs, similar to what was observed in the regular Cu_58_ NC (**Figure** **1**). Our detailed examination revealed that both Cu_58_‐I and Cu_58_‐II exhibit nested Keplerian architectures, featuring Cu_8_ cubic cores (Figure S1, Supporting Information) encircled by four concentric Cu(I) shells (Figure S2, Supporting Information). In both cases, the innermost Cu_6_ octahedron shell is positioned at the center, linking the Cu(I) atoms from each face of the Cu_8_ cubic core. The Cu_24_ rhombicuboctahedron shell shapes the facets of the overall cubic architecture by creating a middle plane with four Cu(I) atoms. Additionally, the Cu_12_ cuboctahedron shell contributes to the edge centers of the overall cubic architecture, while the outermost Cu_8_ cubic shell forms the vertices of the cube. These observations align with our previous findings on Cu_58_ NC.^[^ ^17^ ^]^ Furthermore, we detected three types of ligands (thiolate, PPh_3_ and hydride) arranged in specific geometries to protect these NCs by forming ligand shells. The hydride ligands are positioned interstitially in both cases, connecting the core and the inner shell by forming H_8_ cubic and an H_12_ icosahedron ligand shell geometries (Figure S3, Supporting Information). Despite the change in the carbon chain length, the thiolate ligands in both NCs are arranged similarly into two distinct geometries: an S_12_ icosahedron and an S_24_ truncated cubic ligand shell (Figure S4, Supporting Information). These thiolate ligand shells serve to protect the edges and facets of the cube, ensuring structural stability and integrity. However, a significant change is first visible in the attachment of the PPh_3_ ligands. In the regular Cu_58_ NC, eight PPh_3_ ligands form a complete cubic ligand shell (P_8_) around the NC, which is not observed in Cu_58_‐I and Cu_58_‐II NCs (**Figure** **2**a–c). In Cu_58_‐I NC, we observed one vacant site in the P_8_ ligand shell, where one vertex Cu atom of the Cu_8_ cubic shell is exposed due to this vacancy. In contrast, Cu_58_‐II NC exhibited two vacant sites diagonally in the P_8_ ligand shell, exposing two Cu atoms from the Cu_8_ cubic shell. Since the construction of the P_8_ ligand shell relies on direct Cu—P bond formation involving the Cu(I) atoms from the Cu_8_ cubic shell, we anticipate irregularities in that cationic shell, which may prevent the PPh_3_ ligands from attaching at those specific positions. As we have already discussed the formation of the cationic shells is followed similarly however, we observed a notable geometric distortion in the outermost Cu_8_ cubic shells compared to Cu_58_ NC (Figure 2d,e and Figure S5, Supporting Information). We identified that this outer Cu_8_ cubic shell was formed via thiolate bridging, lacking direct connections to the Cu(I) atoms of the inner shells.^[^ ^17^ ^]^ As a result, we noticed a distinct directional arrangement of the Cu‐S_3_ moiety bridging, forming each vertex of the cube. However, here the distortions arise from the partial dislocation of vertex Cu(I) atoms, disrupting their typical outward directional bonding with the bridging thiolate ligands (Figure 2e,f). Due to this inward distortion of the Cu(I) atom alters the distance between Cu(I) atoms and those forming the adjacent facets contributing from the Cu_24_ rhombicuboctahedron shell. In Cu_58_‐I NC the average distance decreases to 2.665 Å from 3.4108 ± 0.0146 Å (average from the other corners). Similarly, in Cu_58_‐II NC, the average distance decreases to 2.6713 ± 0.0065 Å from 3.4032 ± 0.0173 Å (average from the other corners), indicating strong cuprophilic interactions at the defect sites (Figure S6, Supporting Information). These additional interactions help alleviate the strain from the distortion of the Cu(I) atom at the dislocation sites and stabilize the overall cluster structures. However, this inward orientation of the Cu(I) atom at the vertex sites presents challenges for the attachment of PPh_3_ ligands, likely due to steric crowding. This difficulty in ligand attachment constitutes the primary reason for the observed surface ligand vacancies. Therefore, this vacancy directly stems from the altered bonding environment induced by the distortion of the Cu(I) atoms in the outermost shell.

Further investigation reveals that at each defect vertex site, additional cuprophilic interactions with three adjacent Cu atoms from neighboring facets disrupt the regular geometries of the adjacent shells. More specifically the rhombicuboctahedron shell is formed by connecting eight triangular Cu frames, each maintaining a fixed distance from the vertex Cu atoms of the Cu_8_ cubic core. However, at the defect sites, we observed a change in the interaction between these triangular Cu frames. In the Cu_58_‐I NC, this distance extends to 3.0016 Å, while the average distance at the other ends is 2.8766 ± 0.0319 Å. For the Cu_58_‐II NC, this distance extends to 3.0253 ± 0.0099 Å, with the average distance at the other ends being 2.8876 ± 0.0464 Å (Figure S7, Supporting Information). Additionally, there is an observed alteration in the interatomic distances between these triangular Cu frames and the surrounding vertices of the Cu_8_ cubic core (Figure S8, Supporting Information). We noted a reduction in the average distance at the defect sites compared to the regular sites for both NCs. The shorter interatomic distances indicate an associated inward displacement strain of the triangular Cu frames, generated by the dislocation of the Cu atom in the outermost cationic shell. Consequently, the rhombicuboctahedron shell of both NCs experiences distortions, spreading to the nearby edge centers contributed by the Cu_12_ cuboctahedron shell. We found that the distances between the edge‐center Cu atoms on each constituent vertex can be categorized into two groups. For the defective sides, the distances between the three adjacent edge‐centers are ≈6.2292 ± 0.0004 Å, while the distances between the edge‐centers on the remaining vertices average 5.878 ± 0.0377 Å for Cu_58_‐I NC. For Cu_58_‐II NC, the distances are 6.2411 ± 0.0227 Å and 5.8269 ± 0.0434 Å, respectively (Figure S9, Supporting Information). These observations clearly demonstrate the impact of the distortion of the outer Cu(I) atoms on the inner shell geometry. On the other hand, this surface ligand vacancy directly exposes the defective vertex Cu atoms and indirectly exposes several neighboring facets and edges of these cubic NCs. Notably, the outward orientation of the edge‐center Cu atoms relative to others presents intriguing possibilities, as these edge‐center atoms, along with the vertex Cu atom at the defect sites, may potentially serve as catalytic sites (Figure S10, Supporting Information). However, the distances between these atoms vary because the vertex‐to‐vertex distances change during the formation of the defect‐induced structural architecture, which may uniquely affect their catalytic properties (Figure S11, Supporting Information).

### 2.2 Characterization of NCs and their Stability

**Figure 3:**
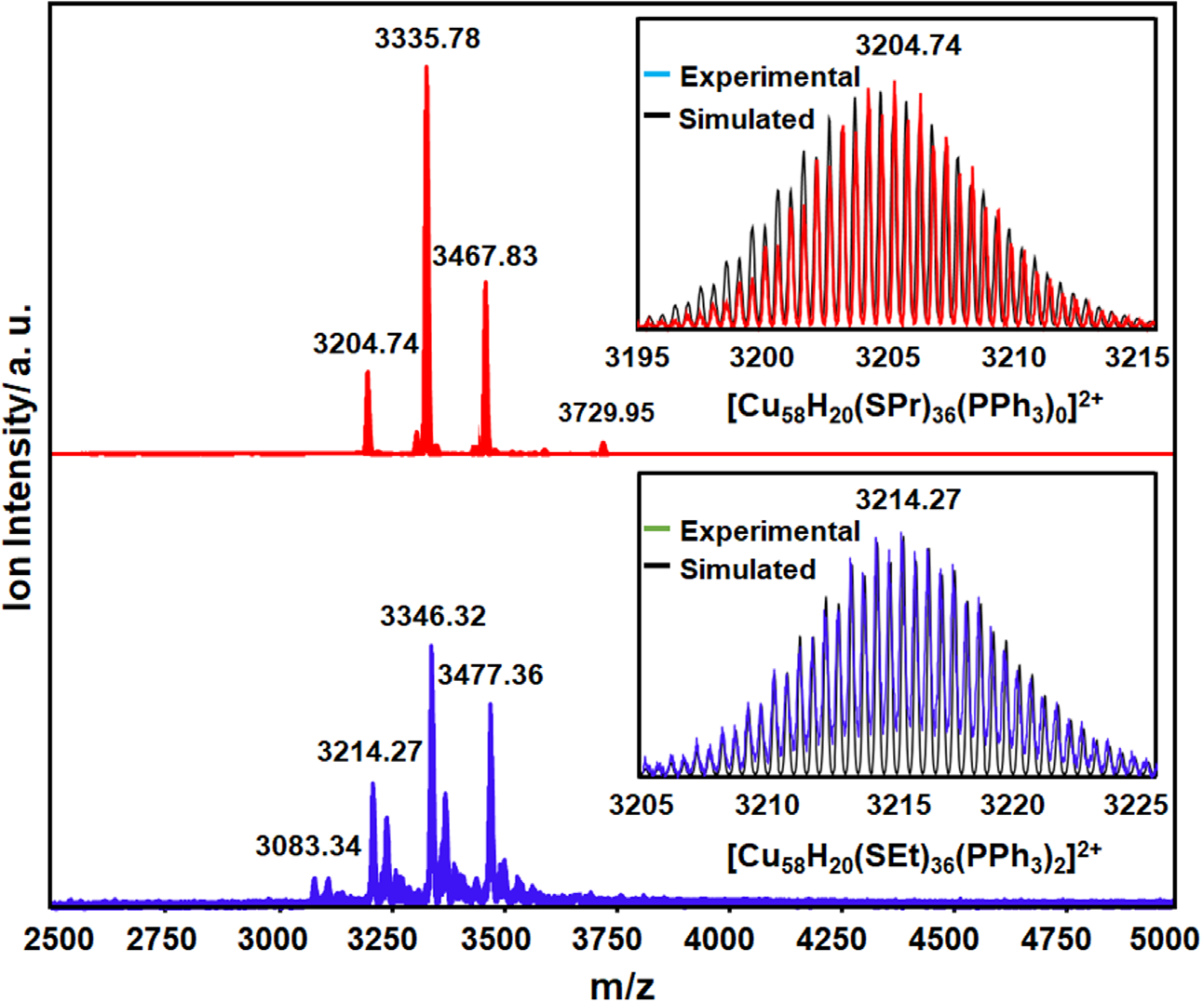
Positive mode ESI mass spectra of Cu_58_‐I and Cu_58_‐II NCs. Inset is showing the matching of experimental and simulated peak of [Cu_58_H_20_(SPr)_36_(PPh_3_)_0_]^2+^ and [Cu_58_H_20_(SEt)_36_(PPh_3_)_2_]^2+^.

In the positive mode electrospray ionization‐mass spectrometry (ESI‐MS), a discernible peak at *m*/*z* = 3204.74 unequivocally affirms the existence of the molecular peak corresponding to [Cu_58_H_20_(SPr)_36_(PPh_3_)_0_]^2+^ (**Figure** **3**). However, achieving the molecular cluster peak featuring all the triphenylphosphine units attached to the cluster node poses considerable challenges due to its dynamic nature.^[^ ^16^, ^18^ ^]^ Additionally, this task becomes even more difficult when defects are present in the structure. Nevertheless, we managed to attribute some of these units, *m*/*z* = 3335.78, 3467.83, and 3729.95 correspond to [Cu_58_H_20_(SPr)_36_(PPh_3_)_1_]^2+^, [Cu_58_H_20_(SPr)_36_(PPh_3_)_2_]^2+^, and [Cu_58_H_20_(SPr)_36_(PPh_3_)_4_]^2+^, respectively in Cu_58_‐I NC. Conversely a different fragmentation pattern is observed in Cu_58_‐II NC where the peaks *m*/*z* = 3083.34, 3214.27, 3346.32 and 3477.36 correspond to [Cu_58_H_20_(SEt)_36_(PPh_3_)_1_]^2+^, [Cu_58_H_20_(SEt)_36_(PPh_3_)_2_]^2+^, and [Cu_58_H_20_(SEt)_36_(PPh_3_)_3_]^2+^ and [Cu_58_H_20_(SEt)_36_(PPh_3_)_4_]^2+^. So, the associated peaks in each cluster confirm the presence of the hydrides and their charge states, along with the different fragmentation patterns based on the defective structural architecture. The stability and dispersibility of the Cu_58_‐I and Cu_58_‐II NC in solution medium are confirmed by transmission electron microscope (TEM) images, which show that their sizes are consistent with those measured by the SCXRD (Figure S12, Supporting Information). Given the highly stable nature of the synthesized NC in the solution medium, we proceeded to examine the effects of such defects on the optical properties. However, no changes were observed in the UV‐vis absorbance spectrum in the solvent medium compared to our previously reported Cu_58_ NC (Figure S13, Supporting Information). So, it can be assumed that the distortion in the vertex Cu(I) atoms and the associated ligand vacancies have no impact on their electronic charge transition.

### 2.3 Preparation of Catalysts and Associated Characterizations

**Figure 4:**
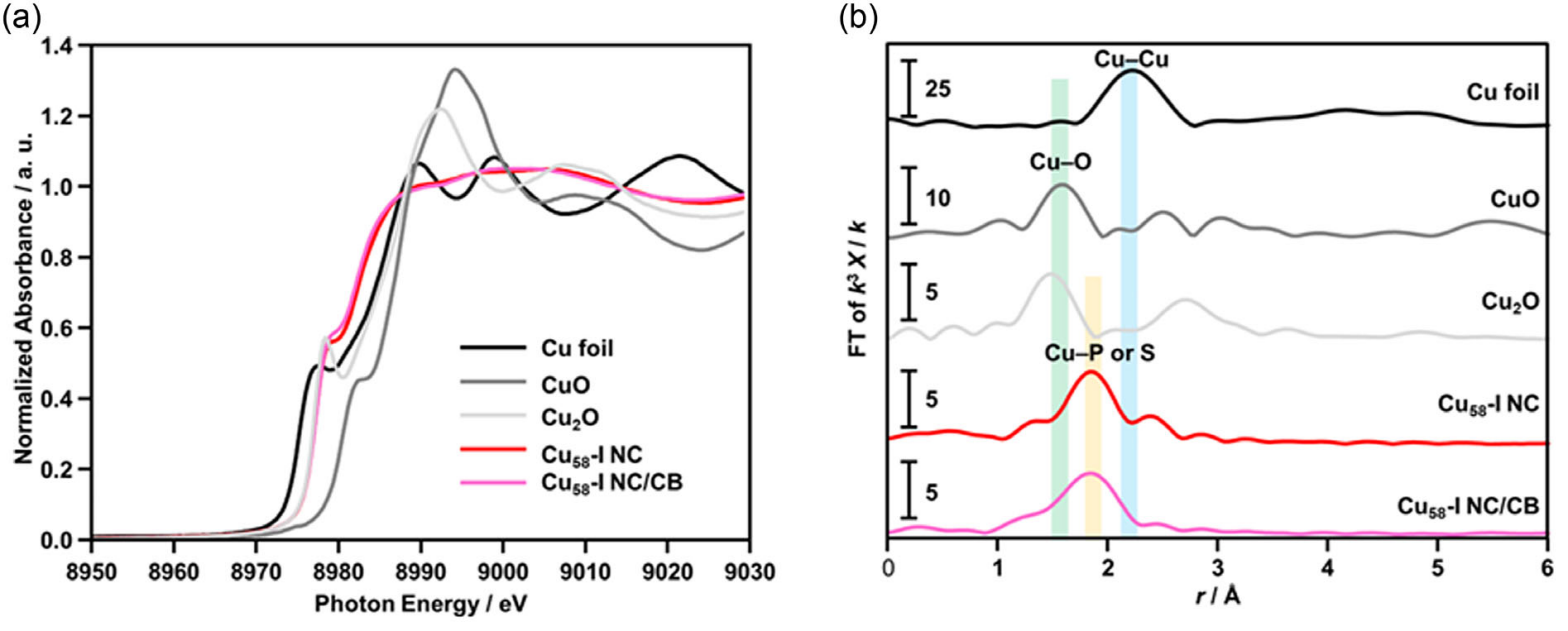
Cu K‐edge a) XANES and b) FT‐EXAFS spectra of Cu_58_‐I NC and Cu_58_‐I NC‐loaded catalysts (Cu_58_‐I NC/CB). In (a,b) spectra of Cu foil, CuO powder and Cu_2_O powder are also shown for comparison.

As per our previous discussions, in our pursuit of the perfect catalyst for electrochemically reducing CO_2_ into a valuable energy feedstock, we aim to utilize the Cu_58_‐I and Cu_58_‐II NCs and evaluate their efficacy against the regular Cu_58_ NC. For the electrochemical evaluation, thin film electrodes were fabricated utilizing the spray coating technique. This involved spray‐casting a slurry of the NC containing Carbon Black (CB) onto a thin carbon paper (CP) substrate as depicted in the experimental section. We confirmed that there was no change in the electronic and geometric structure of Cu_58_‐I and Cu_58_‐II NCs during the loading processes by the X‐ray absorption fine structure (XAFS) spectra (**Figure** **4** and Figure S14, Supporting Information). The X‐ray absorption near‐edge structure (XANES) spectrum at the Cu K‐edge for both the Cu_58_‐I and Cu_58_‐II NCs, as well as the loaded catalysts (Cu_58_‐I NC/CB and Cu_58_‐II NC/CB), demonstrates excellent alignment, indicating the electronic stability of these NCs after loading on CB. In both cases, the pre‐edge peak ≈8980 resembles that of standard Cu_2_O, suggesting the presence of Cu(I) species in all cases (Figure 4a and Figure S14a, Supporting Information). Fourier transforms‐extended X‐ray absorption fine structure (FT‐EXAFS) spectra at the Cu K‐edge reveal a distinct geometric arrangement in the Cu_58_‐I and Cu_58_‐II NCs, indicating a modified ligand environment compared to the standard sample, a characteristic maintained even after loading onto CB (Figure 4b and Figure S14b, Supporting Information). Notably, the peak at ≈1.8 Å in the FT‐EXAFS is attributed to Cu—P or Cu—S bonding in both NCs. Further analysis through R‐space extended X‐ray absorption fine structure (EXAFS) corroborates these observations, highlighting differences from the standard sample spectra while retaining their features upon loading (Figure S15, Supporting Information). Meanwhile, we also confirm the size of these NCs is retained upon loading on the CB surface through the TEM images (Figure S16, Supporting Information).

### 2.4 Electrochemical Performance

**Figure 5:**
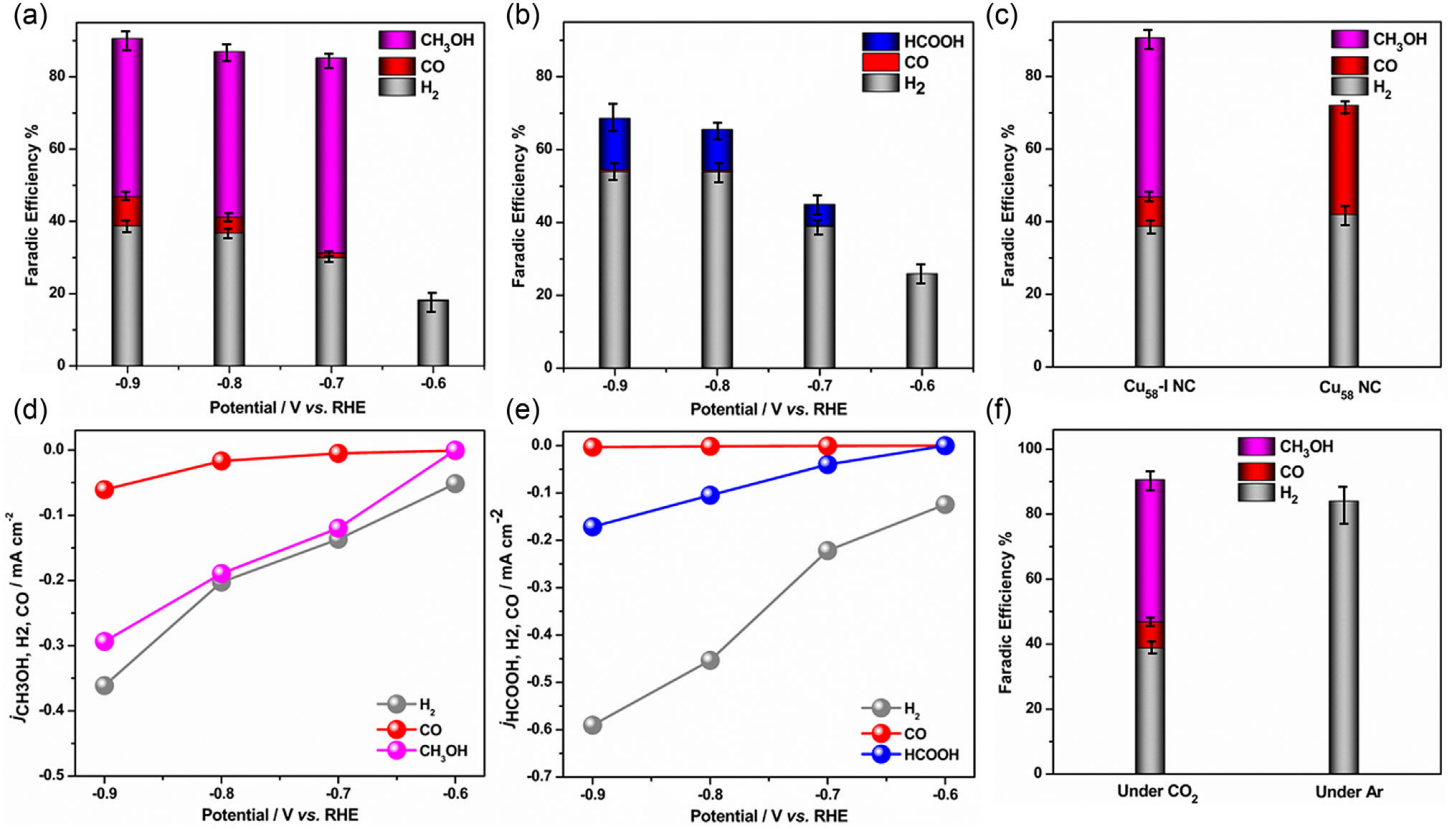
Electrocatalytic performances for CO_2_RR in 0.1 M KHCO_3_ aq. using gas‐flow H‐type cell. a) FE for CO_2_ reduction products for Cu_58_‐I NC‐loaded catalysts at different applied potentials. b) FE for CO_2_ reduction products for Cu_58_‐II NC‐loaded catalysts at different applied potentials. c) Comparisons of FE for Cu_58_‐I NC and Cu_58_ NC‐loaded catalysts at −0.9 V versus RHE. Error bars representing the mean ± SD, *n* = 3. d) Current densities of different products obtained by Cu_58_‐I NC‐loaded catalyst. e) Current densities of different products obtained by Cu_58_‐II NC‐loaded catalyst. f) FE for Cu_58_‐I NC‐loaded catalysts under CO_2_ or Ar flow at −0.9 V versus RHE.

The electrochemical performance of all these NCs was thoroughly evaluated across a range of constant voltages (−0.6 to −0.9 V vs RHE) using CO_2_‐saturated 0.1 M KHCO_3_ aq. as an electrolyte in a gas‐flow H‐type cell setup. Subsequently, a comprehensive analysis employing gas chromatography (GC) and ^1^H NMR was conducted to characterize the gas and liquid products, respectively. The linear sweep voltammetry (LSV) and cyclic voltammetry of these nanoclusters (NCs) under a CO_2_ atmosphere (Figure S17, Supporting Information) showed that Cu_58_‐II NC exhibited the highest reduction current at −0.9 V versus RHE, followed by Cu_58_‐I NC and then Cu_58_ NC. These initial results suggest that corner defect sites are likely responsible for initiating the reduction reaction. Our detailed findings revealed that, despite having similar overall geometrical architectures, these NCs yield different products during the electrocatalytic reduction reaction. We attribute this to variations in the distortion of their cationic shell geometry and surface ligand vacancies, which influence their reactivity by creating preferred catalytic sites. We observed at a potential range of −0.7 to −0.9 V (vs RHE) H_2_, CO and CH_3_OH are the primary products for Cu_58_‐I NC (**Figure** **5**a and Figure S18, Supporting Information) whereas H_2_, CO and HCOOH for Cu_58_‐II NC (Figure 5b and Figure S19, Supporting Information). According to the Faradic Efficiency (FE) analysis, we observed that hydrogen evolution reaction (HER) is dominant (FE_H2_ > 50%) in the case of Cu_58_‐II NC. This dominance is attributed to the higher number of defect sites with more exposed edges and vertices, making the cluster surface easily accessible for the HER reaction. In contrast, Cu_58_‐I NC, which features a single ligand vacancy with limited distortions and exposed sites, selectively produced CH_3_OH with a maximum FE_CH3OH_ of ≈54% at −0.7 V (vs RHE). Comparing this with Cu_58_ NC, which produces only CO as the CO_2_RR product due to its fewer reactive sites, highlights the significant impact of structural architecture on product selectivity and efficiency in CO_2_RR processes (Figure 5c and Figure S20, Supporting Information). The comparison of the associated current densities of the products for both Cu_58_‐I and Cu_58_‐II NCs further displayed their variable reactivities (Figure 5d,e). Especially, the currents of the products based on CO_2_RR were higher for Cu_58_‐I NC (*j*_CH3OH_ and *j*_CO_) than for Cu_58_‐II NC (*j*_HCOOH_ and *j*_CO_), due to the favorable electronic/geometric structure of Cu_58_‐I NC for CO_2_RR. It becomes evident that the structural configuration of Cu_58_‐I NC exhibits a favorable propensity for CH_3_OH selectivity, demonstrating variability across a broad potential range. Notably, as increasingly negative potentials were applied, the selectivity for CH_3_OH in CO_2_RR gradually decreased to ≈44% at −0.9 V, while the production of associated H_2_ and CO increased. This trend became more pronounced at −1.0 V, where FE_CO_ rose to ≈16% and the selectivity for CH_3_OH dropped to ≈37% (Figure S21, Supporting Information). According to the theoretical redox potentials, the formation of CO (−0.10 V vs RHE) and H_2_ (0.00 V vs RHE) requires slightly higher overpotentials compared to methanol formation (0.03 V vs RHE).^[8d]^ These overvoltage differences likely contributed to the decrease in the formation efficiency of methanol (FE_CH3OH_). In addition to the ^1^H NMR detection process, we further confirm the selective production of CH_3_OH, through a GC‐MS measurement. The electrolyte after CO_2_RR exhibited only a peak at the same retention time (r.t. = 5.9 min) as the CH_3_OH introduced as the standard sample, without other CO_2_RR products. This peak showed a pattern with a *m*/*z* of 31, and the simulated fragment pattern based on electron ionization was consistent with that of CH_3_OH (Figure S22, Supporting Information). By achieving the selectivity of CO_2_RR products through precise control of defect‐induced structural architecture, we demonstrated the exceptional catalytic activity of Cu_58_‐I NCs. Notably, Cu_58_‐I NCs are among a limited number of electrocatalysts capable of producing CH_3_OH without concurrent HCOOH generation, underscoring its unique effectiveness.^[^ ^19^ ^]^ However, at higher potentials, the decrease in selectivity may be linked to the loss of key intermediates, which limits the range of products formed. To further elucidate the catalytic properties of these NCs, we investigated them through theoretical calculations in the subsequent section. In our quest to pinpoint the origin of carbon‐based products, we conducted experiments by altering the feedstock source to argon (Ar) gas. Remarkably, under this condition, the detection of only H_2_ as a product suggests that the carbon‐based products originate from the flowed CO_2_ feedstock rather than from impurities or contaminants in the system (Figure 5d). Additionally, we delved into assessing the stability of Cu_58_‐I NC through chronoamperometric measurements conducted at −0.9 V. Intriguingly, the current density remained consistent over an extended period, with no discernible degradation observed for at least 6 h (Figure S23, Supporting Information). This highlights the robust stability of Cu_58_‐I NC under the applied electrochemical conditions, further underscoring its potential as a promising catalyst for sustained CO_2_ reduction reactions.

### 2.5 Density Functional Theory (DFT) Calculations and Mechanistic Insights

**Figure 6:**
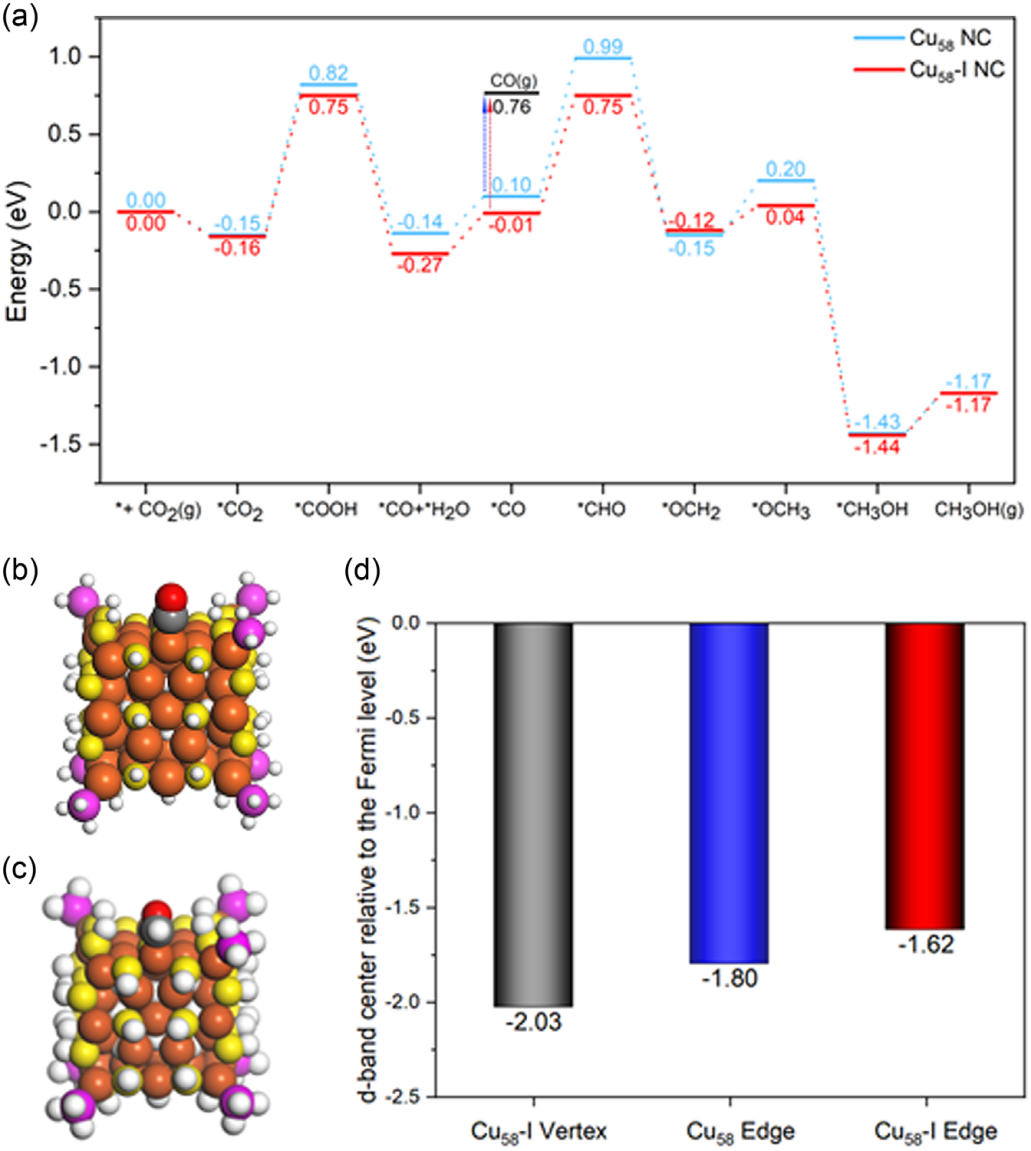
a) DFT‐computed energy profile for CO_2_RR on Cu_58_‐I NC (red) and Cu_58_ NC (blue); b) CO adsorbed on the Cu_58_‐I edge; c) CHO adsorbed on the Cu_58_‐I edge; d) d‐band centers of the different Cu sites on the Cu_58_‐I and Cu_58_ NCs, computed from the local density of states. Color legend: Cu, brown; S, yellow; P, violet; H, white.

To correlate the selectivity difference between Cu_58_ and Cu_58_‐I NCs in electrochemical CO_2_RR with their structural/active‐site difference, we carried out DFT calculations based on the computational hydrogen electrode (see Supporting Information for details). We excluded Cu_5__8_‐II nanoclusters from the study as HER is the main reaction pathway for this nanocluster. Our hypothesis is that at negative enough overpotentials, both catalysts can produce CO_2_ to CO but only Cu_58_‐I NC can further reduce CO to CH_3_OH. To test this hypothesis, we computed the detailed CO_2_RR pathway from CO_2_ to CO and then to CH_3_OH on both Cu_58_ and Cu_58_‐I NCs. As shown in **Figure** **6**a, we found that CO_2_ is weakly adsorbed on both clusters. Then we used the computational hydrogen electrode method and sequentially added H to CO_2_ and computed the intermediate adsorption energies. We found that the formation of *COOH is more favorable than *HCOO (Figure S24, Supporting Information) and less uphill on Cu_58_‐I than Cu_58_. The next step is CO formation from *COOH, which significantly favors Cu_58_‐I over Cu_58_. After H_2_O desorption, *CO is converted to *CHO, which is the potential‐limiting step leading to CH_3_OH formation. On the Cu_58_ NC, CO desorption is easier than *CHO formation, while on the Cu_58_‐I NC, *CHO is competitive against CO desorption. CO and CHO adsorb stronger on the Cu_58_‐I NC than on the Cu_58_ NC. We think that this is a key reason why Cu_58_‐I can catalyze CO_2_RR to CH_3_OH, while Cu_58_ cannot. More interestingly, we found that CO and CHO actually adsorb on the edge Cu site next to the defect Cu site (Figure 6b,c), but on the defect vertex Cu site. In other words, the inward movement of the Cu(I) atom and the missing phosphine ligand at the vertex site rendered the nearby edge Cu sites more reactive. To further understand the intricate connection between the geometric and electronic factors here, we analyzed the local density of states at the different Cu sites (Figure S25, Supporting Information) and found that the d‐band center of the edge site on Cu_58_‐I shifted up in comparison with the edge site on Cu_58_ and the defect vertex site on Cu_58_‐I (Figure 6 d), indicating a higher reactivity.

### 2.6 Sustainability of the NC

The stability of the Cu_58_‐I NC was further confirmed by comparing the size of individual NCs before and after the electrocatalytic reaction within a CB matrix through TEM measurements, which provided insights into its structural integrity (Figure S26, Supporting Information). Furthermore, X‐ray photoelectron spectroscopy (XPS) investigations confirmed the stability of the metal atom valency of this NC both before and after the electrocatalytic reaction (Figure S27, Supporting Information). Consistently, the deconvolution of the binding energies of Cu 2p_3/2_ at 932.8 eV and Cu 2p_1/2_ at 952.6 eV, along with a subtle satellite signal, persisted across the spectrum which indicates the presence of Cu(I) species.

## 3 Conclusion

In summary, herein we reported a novel synthesis method for a surface ligand vacancy‐induced Cu NCs, distinguished by their unique arrangement of metal atoms, deviating from the conventional regular configuration. The structural distortions significantly influence their catalytic behavior, particularly in electrochemical CO_2_RR. Through our electrochemical analyses, we have discovered an exceptional selectivity for CH_3_OH production (FE_CH3OH_ is ≈54% at −0.7 V vs RHE), marking the defect‐induced Cu_58_‐I NC as the first to demonstrate direct electrochemical conversion of such product using NCs. In contrast, the regular Cu_5__8_ NCs primarily produce CO, while the additional defects in the structural architecture promote HER pathways. DFT calculations of the CO_2_RR pathways attributed the selectivity toward CH_3_OH to enhanced binding of CO and CHO intermediates at the edge Cu site next to the vertex Cu site with a ligand vacancy; electronic structure analysis showed that the d‐states of the edge Cu site shifted up due to the geometric distortion induced by the defect and the missing phosphine ligand, rendering the edge Cu site more active. Thus, this comprehensive approach not only elucidates the catalytic mechanism but also underscores the potential of tailored structural designs for enhancing electrochemical CO_2_ reduction efficiency.

## Conflict of Interest

The authors declare no conflict of interest.

## Author Contributions

**Sourav Biswas**: Conceptualization (equal); Investigation (lead); Writing—original draft (lead). **Tomoya Tanaka**: Investigation (equal). **Haohong Song**: Investigation (supporting). **Masaki Ogami**: Investigation (supporting). **Yamato Shingyouchi**: Investigation (supporting). **Sakiat Hossian**: Investigation (supporting). **Maho Kamiyama**: Investigation (supporting). **Taiga Kosaka**: Investigation (supporting). **Riki Nakatani**: Investigation (supporting). **Yoshiki Niihori**: Investigation (supporting). **Saikat Das**: Investigation (supporting). **Tokuhisa Kawawaki**: Conceptualization (lead); Writing—original draft (lead). **De‐en Jiang**: Conceptualization (supporting); Writing—review and editing (equal). **Yuichi Negishi**: Conceptualization (lead); Funding acquisition (lead); Project administration (lead); Resources (lead); Supervision (lead); Writing—review and editing (lead).

## Supporting information

Supplementary Material

## Data Availability

The data that support the findings of this study are available from the corresponding author upon reasonable request.
