# Supplementary material for: Highly Selective Methanol Synthesis Using Electrochemical CO2 Reduction with Defect‐Engineered Cu58 Nanoclusters
Source: Small Sci. 2024 Nov 28;5(2):2400465. doi: 10.1002/smsc.202400465 (PMC11934906; doi:10.1002/smsc.202400465)
Supplement: Supplementary file 1 — Supplementary Material [file SMSC-5-2400465-s001.pdf]

Supporting Information

Highly Selective Methanol Synthesis Using Electrochemical CO_2 Reduction with Defect-Engineered Cu_{58} Nanoclusters

Sourav Biswas,[a] Tomoya Tanaka,[b] Haohong Song,[c] Masaki Ogami,[b] Yamato Shingyochi,[b] Sakiat Hossian,[a] Maho Kamiyama,[b] Taiga Kosaka,[b] Riki Nakatani,[b] Yoshiki Niihori,[a] Saikat Das,[a] Tokuhisa Kawawaki,[d]* De-en Jiang,[e]* Yuichi Negishi[d,f]*

[a] Research Institute for Science & Technology, Tokyo University of Science, 1-3 Kagurazaka, Shinjuku-ku, Tokyo 162-8601, Japan.

[b] Department of Applied Chemistry, Faculty of Science, Tokyo University of Science, 1-3 Kagurazaka, Shinjuku-ku, Tokyo 162-8601, Japan.

[c] Interdisciplinary Materials Science, Vanderbilt University, Nashville, TN 37235, USA.

[d] Carbon Value Research Center, Tokyo University of Science, 2641 Yamazaki, Noda, Chiba 278-8510, Japan.

[e] Department of Chemical and Biomolecular Engineering, Vanderbilt University, Nashville, TN 37235, USA.

[f] Institute of Multidisciplinary Research for Advanced Materials, Tohoku University, Katahira 2-1-1, Aoba-ku, Sendai 980-8577, Japan.

*Corresponding Authors

T.K.: kawawaki@rs.tus.ac.jp

D.J.: de-en.jiang@vanderbilt.edu

Y.N.: negishi@rs.tus.ac.jp; yuichi.negishi.a8@tohoku.ac.jp

Table of Contents

Name	Description	Page No.
	Experimental Section	S3-S7
Table S1	Crystal data and structure refinement parameters of Cu_{58}-I NC	S8
Table S2	Crystal data and structure refinement parameters of Cu_{58}-II NC	S9
Figure S1	Core geometry of Cu_{58}-I and Cu_{58}-II NCs	S10
Figure S2	Cationic shell geometry of Cu_{58}-I and Cu_{58}-II NCs	S10
Figure S3	Hydride shell geometry of Cu_{58}-I and Cu_{58}-II NCs	S11
Figure S4	Thiolate shell geometry of Cu_{58}-I and Cu_{58}-II NCs	S11
Figure S5	Geometry distortion in Cu_8 cubic shells	S12
Figure S6	Additional three Cu-Cu interaction due to the distortion	S12
Figure S7	Interaction changes in triangular Cu frame of Cu_{24} shell geometry	S13
Figure S8	The associated distortion in Cu_{24} shell geometries	S13
Figure S9	Distortion in Cu_{12} shell geometries	S14
Figure S10	The exposed facets and edges due to the surface ligand vacancy	S14
Figure S11	The diagonal distances of the vertices of different NCs	S15
Figure S12	TEM image of Cu_{58}-I and Cu_{58}-II NCs	S15
Figure S13	UV-vis absorbance spectrum in the solvent medium	S16
Figure S14	Cu K-edge XANES and FT-EXAFS spectra of Cu_{58}-II NC with or without CB	S16
Figure S15	Cu K-edge EXAFS spectra of Cu_{58}-II NC with or without CB	S17
Figure S16	TEM images of catalysts after loading on CB	S17
Figure S17	LSV and CV data for electrocatalytic CO_2RR results	S18
Figure S18	Raw data for electrocatalytic CO_2RR results for Cu_{58}-I NC-loaded catalysts	S19
Figure S19	Raw data for electrocatalytic CO_2RR results for Cu_{58}-II NC-loaded catalysts	S20
Figure S20	Raw data for electrocatalytic CO_2RR results for Cu_{58} NC-loaded catalysts	S21
Figure S21	FE for CO_2 reduction products for Cu_{58}-I NC-loaded catalysts	S22
Figure S22	Results of GC-MS analysis of electrocatalytic CO_2RR products	S22
Figure S23	Stability tests of the Cu_{58}-I NC-loaded catalysts for CO_2RR at −0.9 V vs. RHE	S23
Figure S24	DFT-calculated energy profiles for CO_2 reduction reactions on Cu_{58}-I NC, leading to two different products (HCOOH and CH_3OH)	S23
Figure S25	Projected density of states for the *d*-states of the different Cu sites on the Cu_{58}-I and Cu_{58} NCs	S24
Figure S26	TEM image of Cu_{58}-I NC-loaded catalysts after electrocatalytic CO_2RR	S24
Figure S27	XPS spectra of Cu_{58}-I NC-loaded catalyst after electrocatalytic CO_2RR	S25
	References	S26

Experimental Section:

Materials characterization

Electrospray ionization mass spectrometry was performed using a reflectron-type time of flight MS system (Bruker, microTOF II). The NCs were dissolved in a mixture of chloroform and acetonitrile. The isotope distribution was calculated using an isotope pattern simulator (JEOL, Isotope Pattern Simulator). X-ray photoelectron spectroscopy (XPS) experiments were conducted on a JPS-9-1-MC electron spectrometer (JEOL, Tokyo, Japan) utilizing the Mg-Kα line (1253.6 eV) as the excitation source. All the binding energies were referenced to the neutral C 1s peak at 284.6 eV. UV-Vis absorption spectra were acquired using a JASCO V-770 spectrophotometer. The transmission electron microscope (TEM) images were recorded with a H-9500 electron microscope (HITACHI, Tokyo, Japan) or JEM-2100 electron microscope (JEOL, Tokyo, Japan) operating at 200 kV, typically using magnification of 600,000. X-ray absorption fine structure (XAFS) measurements were performed at beamline BL01B1 of the SPring-8 facility of the Japan Synchrotron Radiation Research Institute (proposal numbers 2022B1823, 2023A1675 and 2023B1825). The incident X-ray beam was monochromatized with a Si(111) double-crystal monochromator. Cu K-edges of XAFS spectra of all samples (as well as Cu foil, Cu_2O powder, and CuO powder as a reference) were recorded in transmission mode with ionization chambers. The X-ray energies for the Cu K-edges were calibrated with Cu foil, respectively. X-ray absorption near-edge structure (XANES) and extended XAFS (EXAFS) spectra were analyzed with xTunes[S1] as follows. The χ spectra were extracted by subtracting the atomic absorption background by cubic spline interpolation and normalized to the edge height. The normalized data were used as the XANES spectra. The k^3-weighted χ spectra in the k range 3.0–12.0 Å^{-1} for the Cu K-edges were Fourier-transformed into r space for structural analysis.

Synthesis of Cu_{58}-I NC

In accordance with our previous approach to synthesizing Cu_{58} NC, we replicated the procedure with slight modifications to the ligand compositions.[S2] 0.16 mmol of $Cu(CH_3CN)_4BF_4$ and 0.1 mmol of PPh_3 were dissolved in the mixture solution of 2 mL acetonitrile and 0.5 mL chloroform at room temperature which produced a colorless solution. After 5 min of stirring, 0.1 mmol of HSPr was added to the reaction mixture and continued

stirring. After that 1 mmol $NaBH_4$ dissolved in 2.5 mL methanol, was added into mixture at once under at room temperature and the color of the solution becomes red from colorless. The reaction was kept for another 1 hour under continuous stirring. After completion, the reaction mixture was centrifuged, and the red precipitate was obtained. Red precipitated was then washed with methanol/acetonitrile (volume ratio 3:1) for three times and kept for drying overnight. The precipitate was then dissolved in the solvent mixture of chloroform/hexane (volume ratio 1:1). The final clear solution was kept for crystallization at ambient conditions. After 5-6 days, red, block-shaped crystals were obtained.

Synthesis of Cu_{58}-II NC

We synthesized the Cu_{58}-II NC by following a similar method as before, substituting the thiol with 0.12 mmol of HSEt, and conducting the reaction in an ice bath. The final red precipitate was dissolved in solvent mixture of methanol/acetonitrile (volume ratio 1:3). The solution was left to crystallize, yielding red, block-shaped crystals after 7 days.

Methods of DFT calculations

Spin-polarized DFT calculations were performed using the Vienna ab initio Simulation Package (VASP).[S3, S4] The Perdew-Burke-Ernzerhof (PBE)[S5] functional of generalized-gradient approximation (GGA) was used for the electron exchange and correlation. The electron-core interaction was described using the projector-augmented wave method (PAW). The Grimme's DFT+D3 method was used to account for van der Waals interactions.[S6] The kinetic energy cutoff was set to 500 eV for the plane wave basis set, and the Brillouin zone was sampled with the gamma point only. $[Cu_{58}H_{20}(SPr)_{36}(PPh_3)_7]^{2+}$ was simplified as $[Cu_{58}H_{20}(SH)_{36}(PH_3)_7]^{2+}$ which was placed in the centre of a large cubic box of 25.50Å×25.50Å×25.50 Å.

Catalyst preparation

To prepare Cu_{58} NCs loaded electrocatalysts (Cu_{58} NCs/CB), Cu_{58} NCs were loaded onto the CB using the impregnation method (Scheme S1). Specifically, Cu_{58} NCs crystal were dissolved in chloroform, and the concentration was measured by ICP-MS. Then the NC solution was added to the CB. In this process, the Cu loading ratio was set to 10 wt%. The

mixture was mixed at room temperature until the solvent evaporated. Finally, Cu_{58} NCs/CB was obtained by evacuating overnight in a desiccator. A similar method was used to prepare the Cu_{58}-I NC loaded electrocatalysts (Cu_{58}-I/CB) and Cu_{58}-II NC loaded electrocatalysts (Cu_{58}-II/CB).

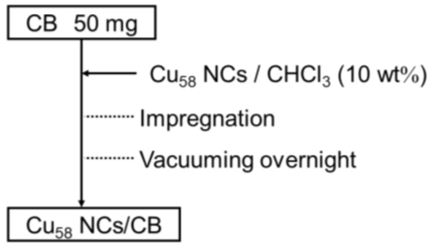

Scheme S1. Preparation method of Cu_{58} NC/CB. A similar method was used to prepare Cu_{58}-I/CB and prepare Cu_{58}-II/CB.

Electrochemical measurement

All electrochemical measurements for the CO_2RR were performed with an ECstat-302 (EC FRONTIER, Japan) with a flow cell (EC FRONTIER, Japan). First, to prepare the catalyst slurry, Cu catalyst (10.1 mg) was added to a solution consisting of ultrapure water (2 mL), 2-propanol (0.5 mL), and Nafion® solution (10 μL). The obtained mixture was sonicated in an ice-water bath for 30 min to disperse the Cu catalyst (Cu_{58} NCs/CB, Cu_{58}-I NCs/CB and Cu_{58}-II NCs/CB) and afford a catalyst slurry. Then, the catalyst slurry (1.1 mL) was sprayed on carbon paper (SIGRACET®GDL 22BB) (φ = 2 cm), which was used as the working electrode. A Pt mesh electrode was used as the counter electrode. A silver/silver chloride (Ag/AgCl) electrode was used as the reference electrode. Each electrode was set in an electrochemical measurement system containing 0.1 M $KHCO_3$ (pH = ~7 under CO_2 sat.) as the electrolyte. In the measurements, Ar gas was bubbled for 15 min and then cyclic voltammetry (CV) was conducted for cleaning the electrodes. After CV, Chronoamperometry (CA) was performed under CO_2 (flow rate: 15 mL min^{-1}) at –0.6 V (vs. RHE) for 30 min. After that, the electrolyte is replaced and Ar bubbling is performed for the next CA. The detailed scheme is shown in Scheme S2. Gas products were analyzed by online gas chromatograph (Shimadzu, GC-8A, TCD or FID; Ar or N_2 carrier gas, respectively). Liquid products were analyzed by ^{1}H NMR spectroscopy. Typically, 200 μL of electrolyte after electrolysis was mixed with 200 μL of D_2O containing 0.02 μL of DMSO as internal

standard. In GC-MS measurements, liquid products were analyzed with capillary column (SH-RTx-Wax; Restek) under He carrier gas (Shimadzu, GCMS-QP2010SE).

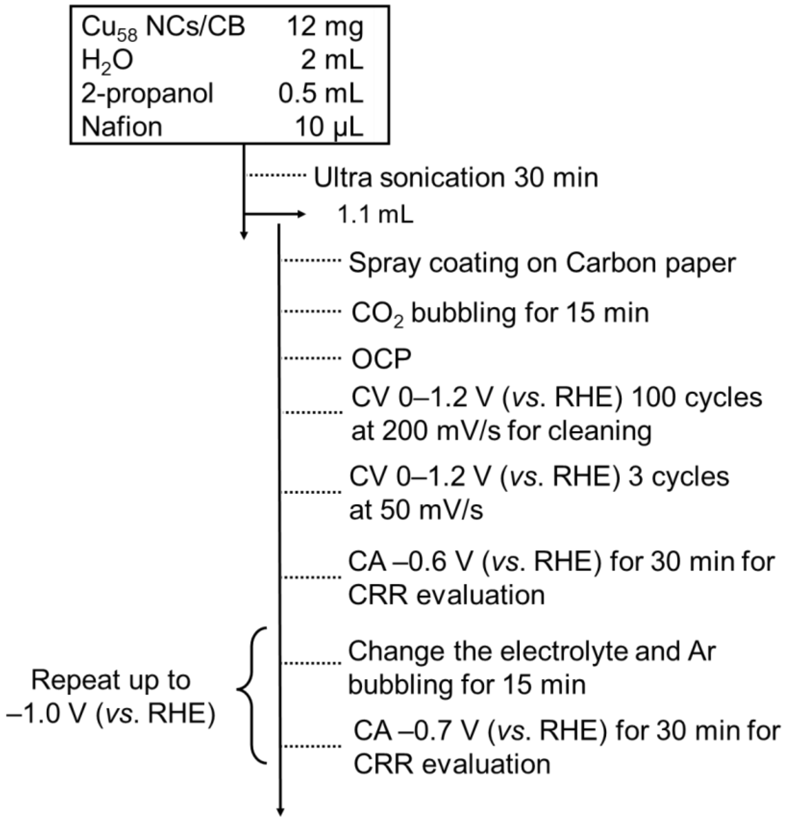

Scheme S2. Preparation method of working electrode and electrochemical measurement protocol for CO_2RR.

X-ray crystallography details

A single crystal was immersed in the cryoprotectant Parabar 10312 (Hampton Research, 34 Journey, Aliso Viejo, CA 92656-3317 USA) and kept at 90 K during diffraction data collection. A Bruker D8 QUEST diffractometer was used to collect the diffraction data for the single crystal using monochromated Mo Kα radiation (λ = 0.71073 Å). Although many crystals from different batches were checked for the diffraction experiment, all of them lacked higher angle data. However, the collected diffraction data was good enough to obtain a structure containing Cu(I) ions, S, P, and a few C atoms, which was solved by SHELXT[S7] using the intrinsic phasing method in Apex3 Bruker Software Suite.[S8] Later, during refinement the full crystal structure was completed using the full-matrix least squares method

against F2 by SHELXL-2018/3 in Olex2 GUI[S9] All the atoms including propylthiolates, n-heaxane solvent, and $[BF_4]^-$ anions were refined anisotropically. A few disordered phenyl rings were fixed by AFIX 66. We also assigned the hydrides (H^-) based on the q peak, and again confirmed by ESI-MS.

For the Cu_{58}-II NC, we could not assign the full $[BF_4]^-$ anions and instead we partially assigned a $[BF_3]$ fragment. To further confirm the anion component of the Cu_{58}-II NC, we analysed the negative mode ESI spectrum that confirms the presence of $[BF_4]^-$ as anions through its peak at *m/z* = 86.9266 (attached below).

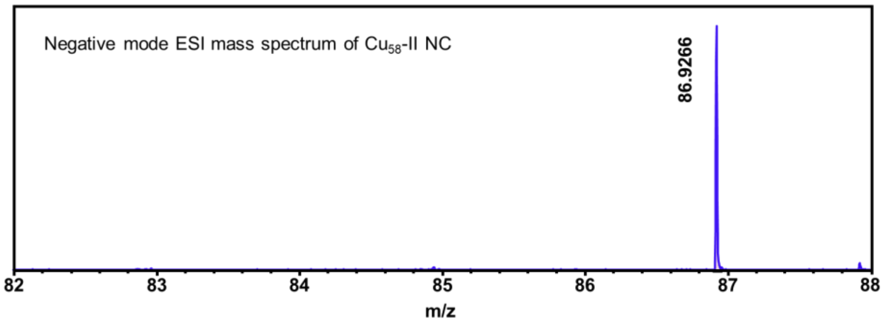

Statistical analysis

We included error bars in the Faradic efficiency (FE) plots, with each error bar representing the standard deviation (SD) from the mean of three experimental measurements (n=3).

Table S1. Crystal data and structure refinement parameters of Cu_{58}-I NC.

Identification code	Cu_{58}-I
Empirical formula	$C_{243}H_{395}B_2Cu_{58}F_8P_7S_{36}$
CCDC number	20230609
Formula weight	8546.36
Temperature/K	100.15
Crystal system	trigonal
Space group	*P-3*
a/Å	23.3506(16)
b/Å	23.3506(16)
c/Å	35.229(4)
α/°	90
β/°	90
γ/°	120
Volume/Å^3	16635(3)
Z	2
ρ_{calc}/g cm^{-3}	1.706
μ/mm^{-1}	3.919
F(000)	8596.0
Crystal size/mm^3	0.3 × 0.1 × 0.1
Radiation	MoKα (λ = 0.71073)
2Θ range for data collection/°	3.468 to 30.546
Index ranges	$-17 \le h \le 17$, $-17 \le k \le 17$, $-26 \le l \le 26$
Reflections collected	77528
Independent reflections	4745 [R_{int} = 0.1983, R_{sigma} = 0.0636]
Data/restraints/parameters	4745/962/1060
Goodness-of-fit on F^2	1.162
Final R indexes [I>=2σ (I)]	R_1 = 0.0473, wR_2 = 0.1207
Final R indexes [all data]	R_1 = 0.0881, wR_2 = 0.1579
Largest diff. peak/hole / e Å^{-3}	0.89/-0.46

Table S2. Crystal data and structure refinement parameters of Cu_{58}-II NC.

Identification code	Cu_{58}-II
Empirical formula	$C_{90}H_{145}Cu_{29}P_3S_{18}BF_3$
CCDC number	2373284
Formula weight	3807.84
Temperature/K	90.15
Crystal system	triclinic
Space group	P-1
a/Å	19.1420(12)
b/Å	19.3505(12)
c/Å	22.3362(13)
α/°	93.148(2)
β/°	98.192(2)
γ/°	105.219(2)
Volume/Å^3	7864.9(8)
Z	2
ρ_{calc}/g cm^{-3}	1.608
μ/mm^{-1}	4.127
F(000)	3782
Crystal size/mm^3	0.1 x 0.06 x 0.05
Radiation	MoKα (λ = 0.71073)
2Θ range for data collection/°	2.204 to 27.606
Index ranges	$-20 \leq h \leq 22$, $-21 \leq k \leq 23$, $-26 \leq l \leq 26$
Reflections collected	76675
Independent reflections	27331 [R_{int} = 0.0952, R_{sigma} = 0.1189]
Data/restraints/parameters	27331/53/1261
Goodness-of-fit on F^2	1.162
Final R indexes [I>=2σ (I)]	R_1 = 0.0635, wR_2 = 0.1408
Final R indexes [all data]	R_1 = 0.1149, wR_2 = 0.1629
Largest diff. peak/hole / e Å^{-3}	1.747/-1.109

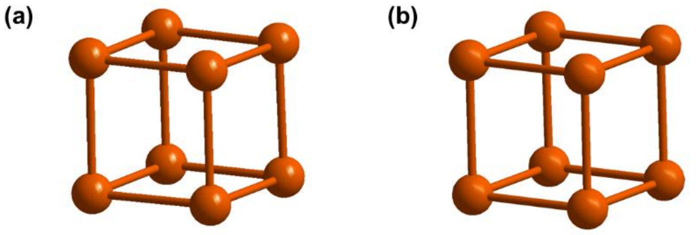

Figure S1. Cu_8 cubic core of (a) Cu_{58}-I NC and (b) Cu_{58}-II NC. Color legend: Cu, brown.

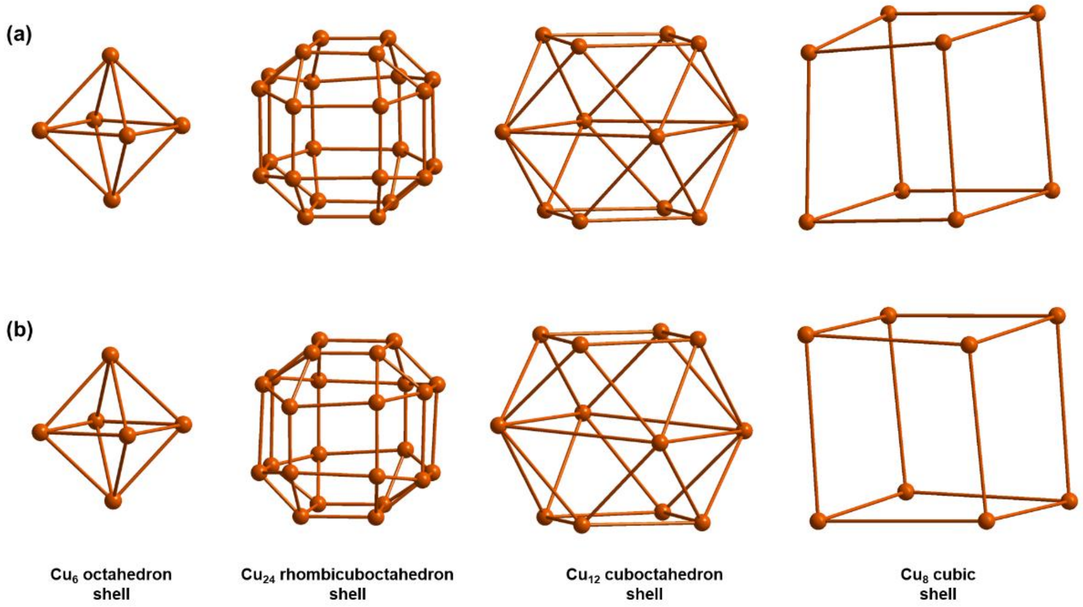

Figure S2. Four concentric Cu(I) cationic shells of (a) Cu_{58}-I NC and (b) Cu_{58}-II NC. Color legend: Cu, brown.

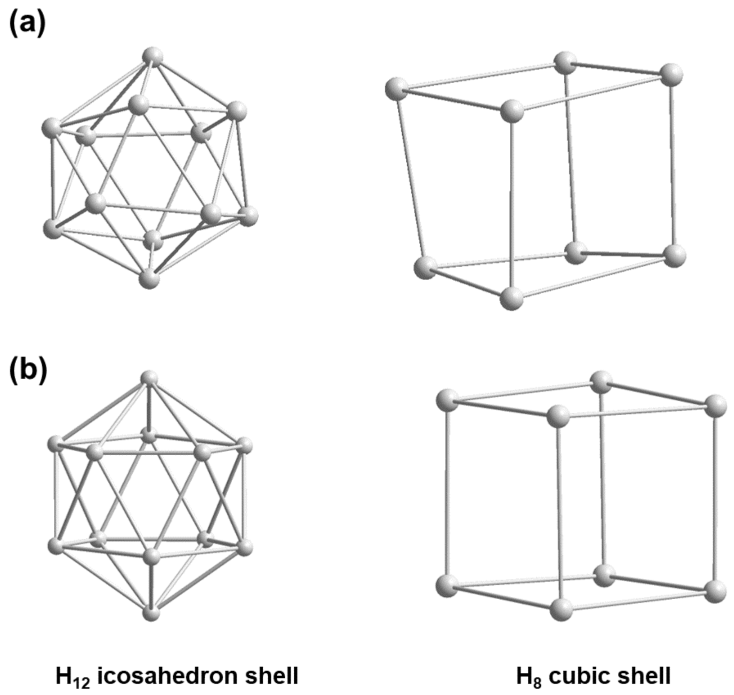

Figure S3. Hydride ligands are attached on the core by forming two geometric architectures in (a) Cu_{58}-I NC and (b) Cu_{58}-II NC Color legend: H, white.

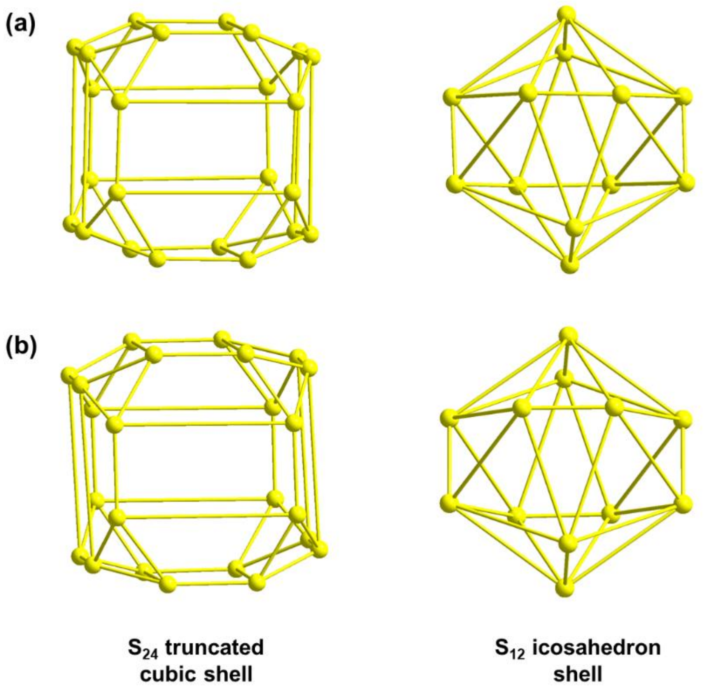

Figure S4. Thiolate ligands are forming two geometric architecture which are bridging all the Cu atoms in (a) Cu_{58}-I NC and (b) Cu_{58}-II NC. Color legend: S, yellow.

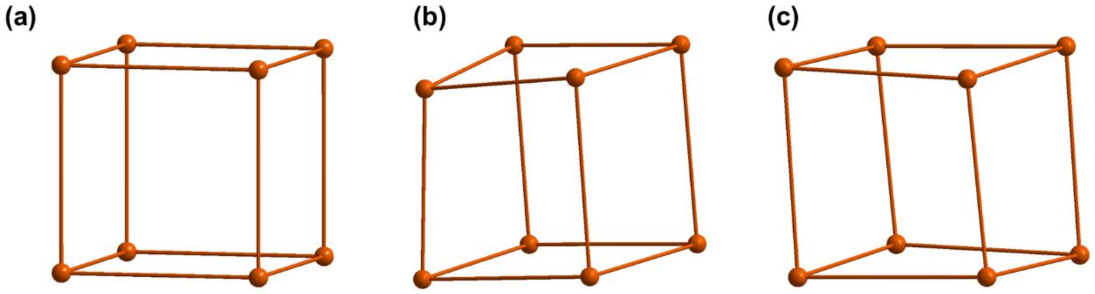

Figure S5. Geometric distortion in Cu_8 cubic shell of (a) Cu_{58} NC, (b) Cu_{58}-I NC and (c) Cu_{58}-II NC. Color legend: Cu, brown.

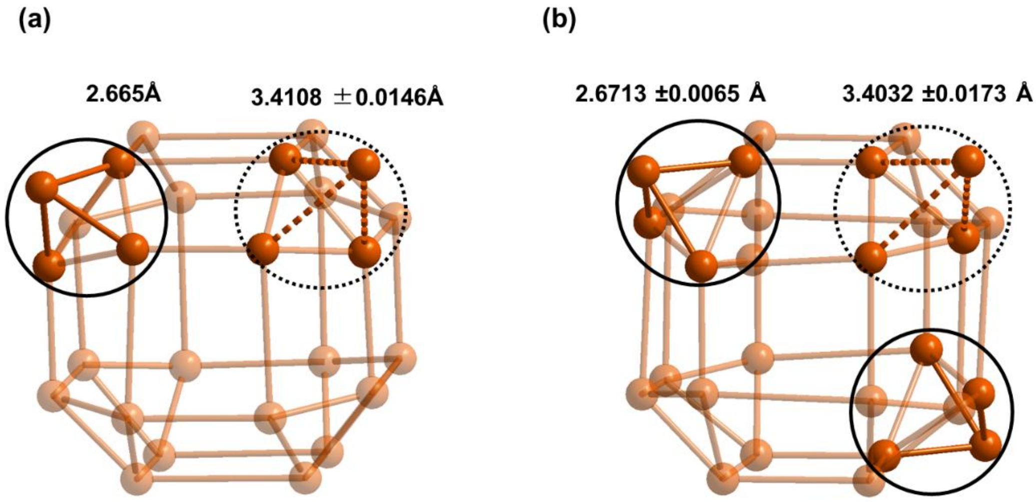

Figure S6. Additional three Cu-Cu interaction due to the distortion of one vertex Cu atom on the other side showing the average distance of the Cu atom from the Cu_8 cubic shell (a) Cu_{58}-I NC and (b) Cu_{58}-II NC. Black complete circles indicate the defect sites and the dotted circle indicate the regular sites. We calculated the average Cu-Cu distances form all direction with similar environment. But for clear pictorial presentation we showed the dotted circle in one position only. Color legend: Cu, brown.

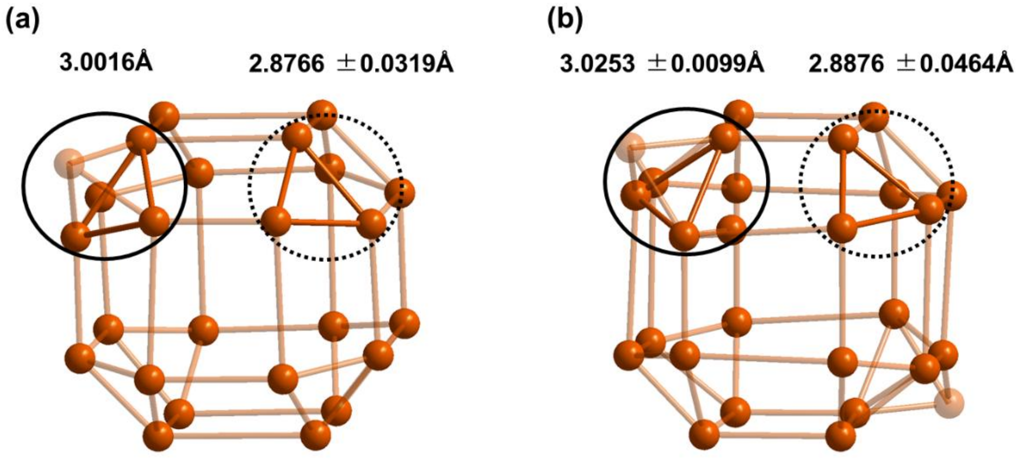

Figure S7. Interaction changes in triangular Cu frame of Cu_{24} rhombicuboctahedron shell geometry which results inward displacement of the three Cu(I) atoms constituent of the defect vertex sites of (a) Cu_{58}-I NC and (b) Cu_{58}-II NC. Black complete circles indicate the defect sites and the dotted circle indicate the regular sites. We calculated the average Cu-Cu distances form all direction with similar environment. But for clear pictorial presentation we showed the dotted circle in one position only. Color legend: Cu, brown.

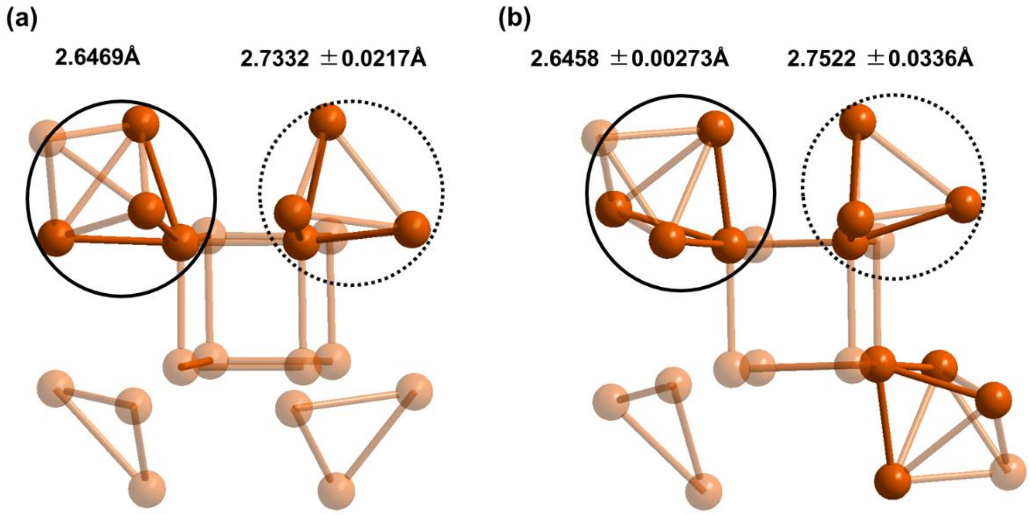

Figure S8. The associated distortion in Cu_{24} rhombicuboctahedron shell geometry which results inward displacement of (a) Cu_{58}-I NC and (b) Cu_{58}-II NC. Black complete circles indicate the defect sites and the dotted circle indicate the regular sites. We calculated the average Cu-Cu distances form all direction with similar environment. But for clear pictorial presentation we showed the dotted circle in one position only. Color legend: Cu, brown.

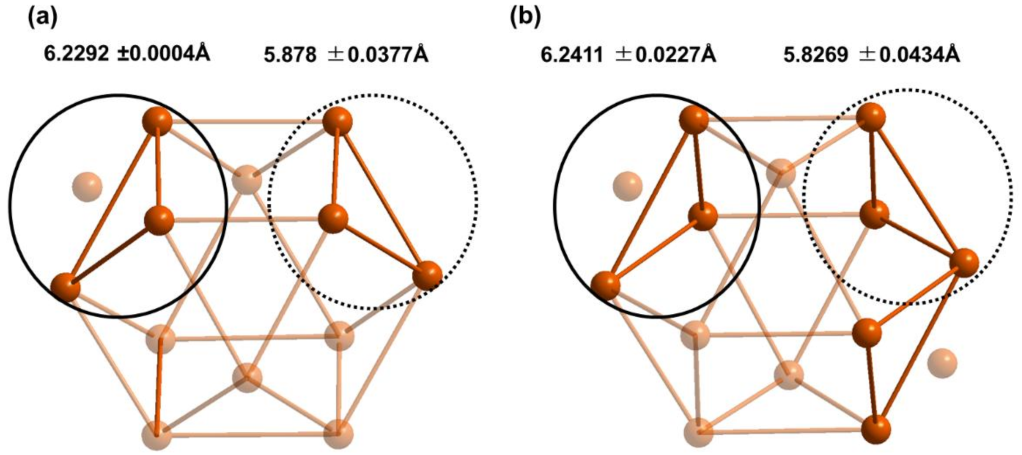

Figure S9. The associated distortion in Cu_{12} cuboctahedron shell geometry with the different interatomic distances of (a) Cu_{58}-I NC and (b) Cu_{58}-II NC. Black complete circles indicate the defect sites and the dotted circle indicate the regular sites. We calculated the average Cu-Cu distances form all direction with similar environment. But for clear pictorial presentation we showed the dotted circle in one position only. Color legend: Cu, brown.

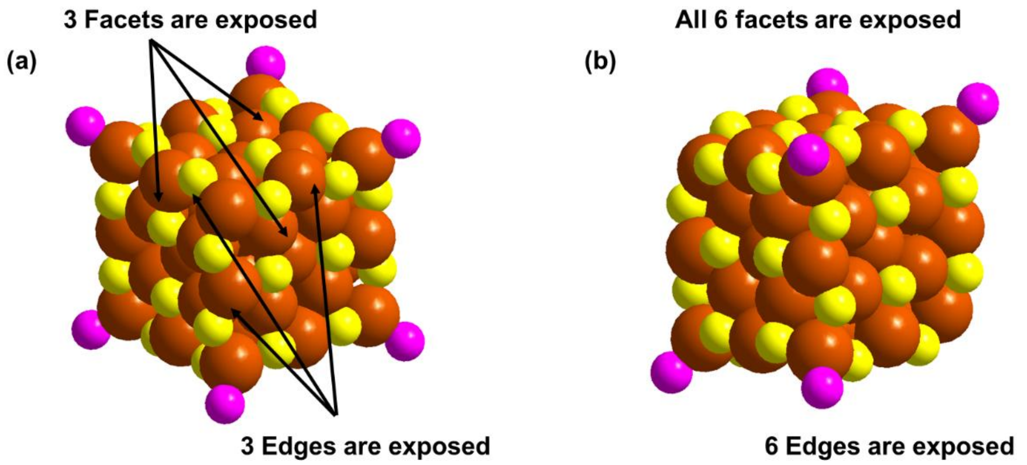

Figure S10. The exposed facets and edges due to the surface ligand vacancy of (a) Cu_{58}-I NC and (b) Cu_{58}-II NC. Color legend: Cu, brown; S, yellow; P, violet.

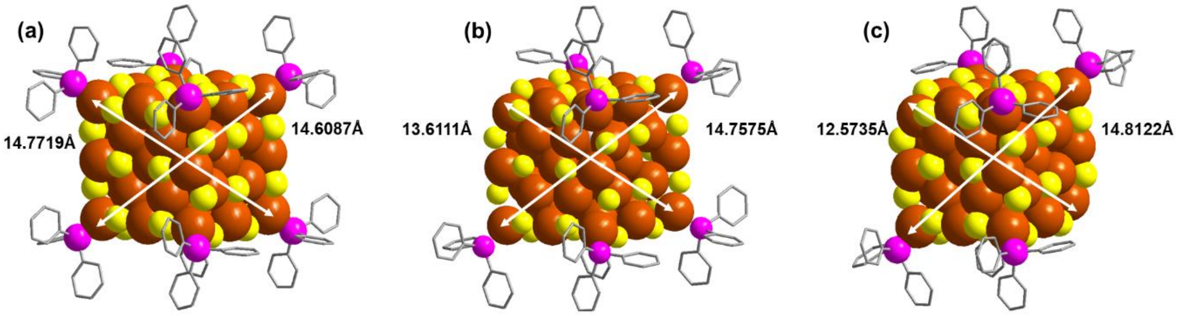

Figure S11. The diagonal distances of the vertices of (a) Cu_{58} NC, (b) Cu_{58}-I NC and (c) Cu_{58}-II NC. Color legend: Cu, brown; S, yellow; P, violet; C, grey stick.

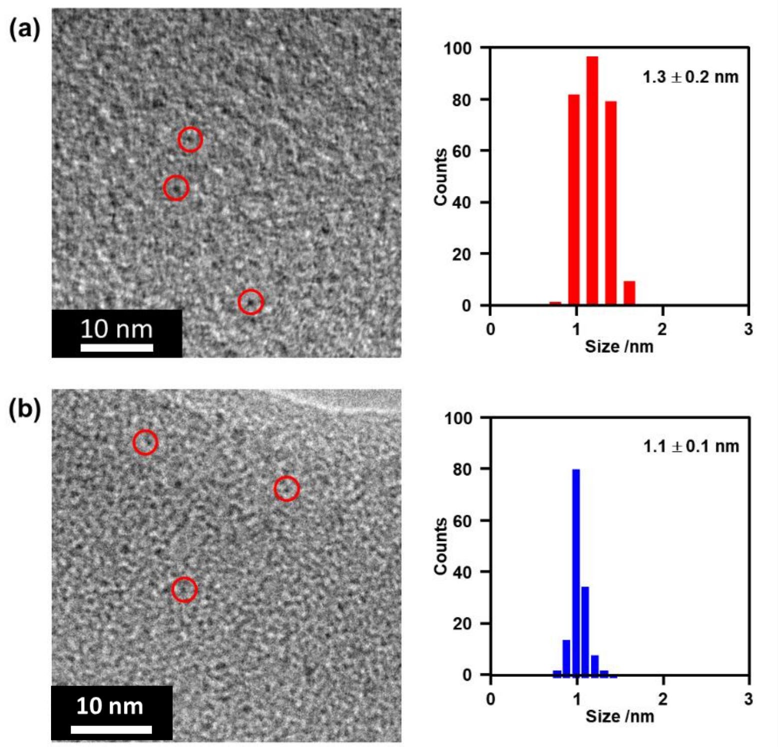

Figure S12. TEM image of (a) Cu_{58}-I NC and (b) Cu_{58}-II NC. Red circles are identifying some of the cluster units.

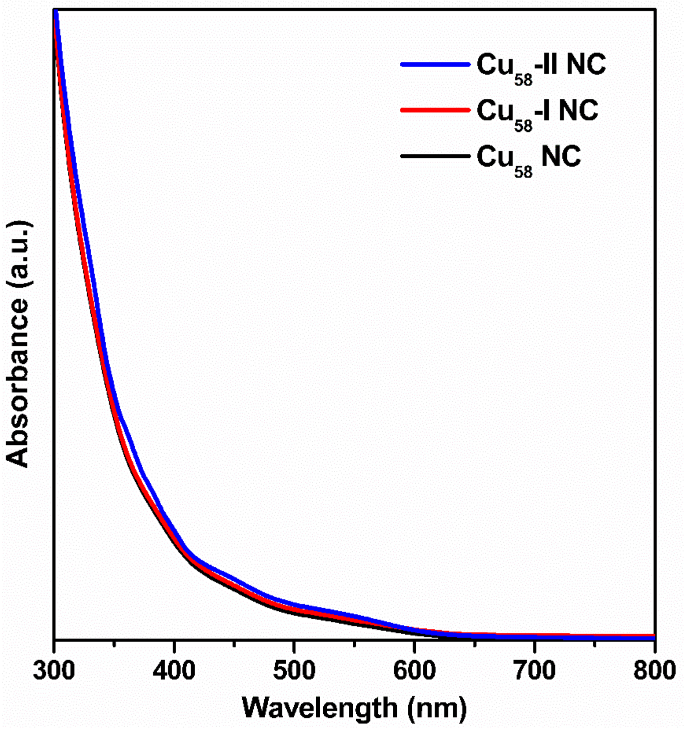

Figure S13. UV-vis absorbance spectrum in the solvent medium of Cu_{58}, Cu_{58}-I and Cu_{58}-II NCs.

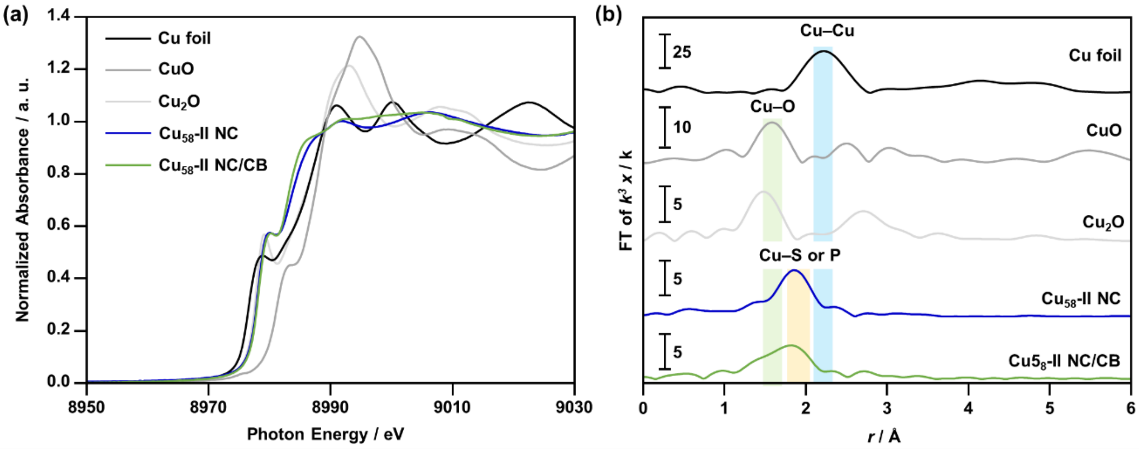

Figure S14. Cu K-edge (a) XANES and (b) FT-EXAFS spectra of Cu_{58}-II NC and Cu_{58}-II NC-loaded catalysts (Cu_{58}-II NC/CB). In (a) and (b), spectra of Cu foil, CuO powder and Cu_2O powder are also shown for comparison.

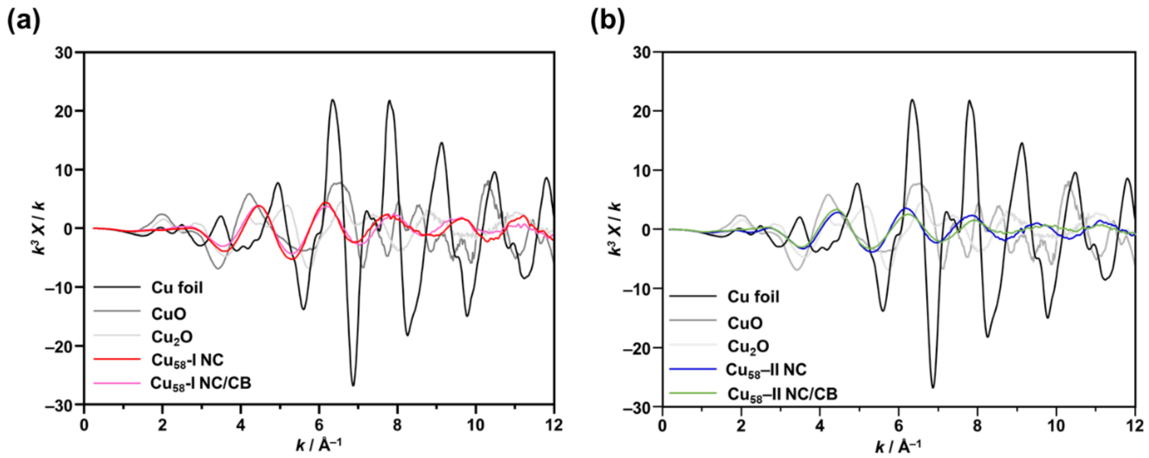

Figure S15. Cu K-edge EXAFS spectra of (a) Cu_{58}-I NC and Cu_{58}-I NC-loaded catalysts (Cu_{58}-I NC/CB) and (b) Cu_{58}-II NC and Cu_{58}-II NC-loaded catalysts (Cu_{58}-II NC/CB). The EXAFS spectra of Cu foil, CuO powder and Cu_2O powder are also shown for comparison.

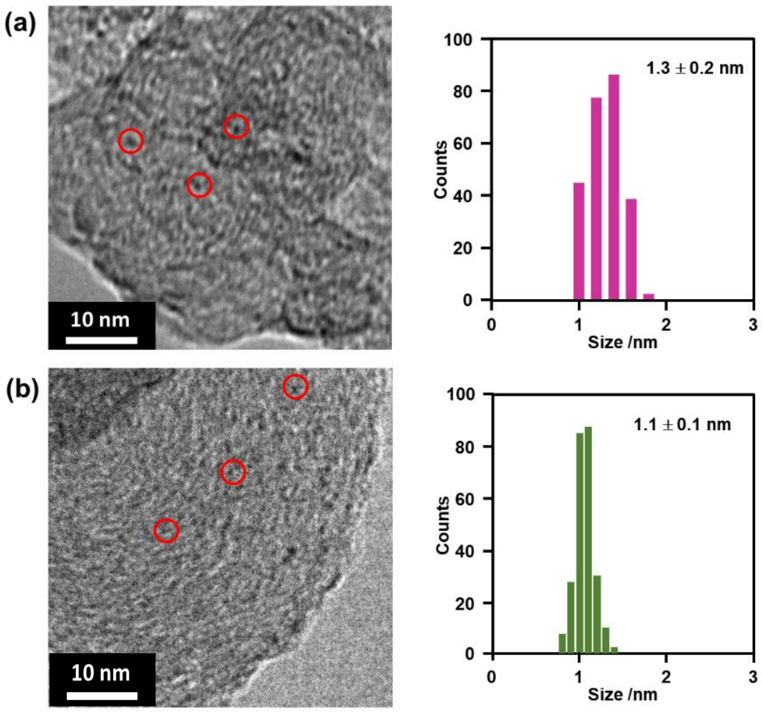

Figure S16. TEM images of (a) Cu_{58}-I/CB and (b) Cu_{58}-II/CB NCs. Red circles are identifying some of the cluster units.

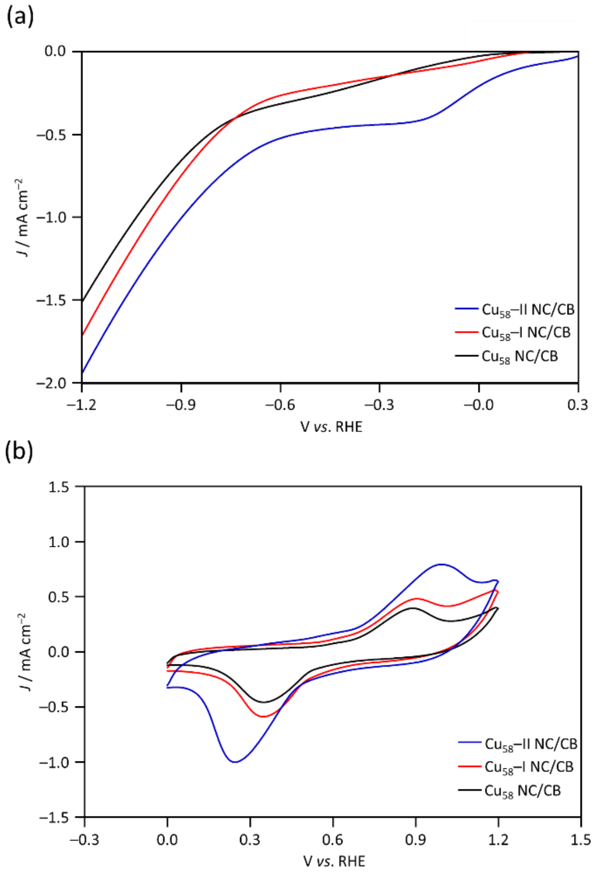

Figure S17. The raw data for electrocatalytic CO_2RR results. (a) LSV and (b) CV in 0.1 M $KHCO_3$ aq. under CO_2 flow on Cu_{58}-II, Cu_{58}-I and Cu_{58} NC-loaded catalysts.

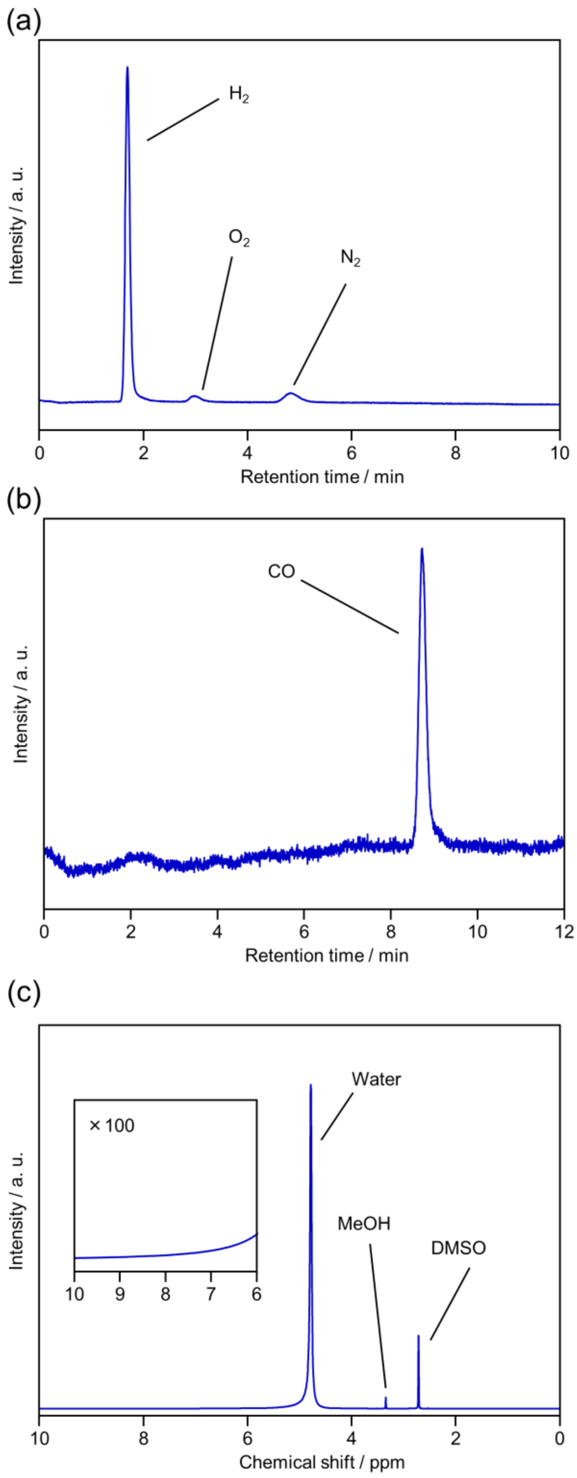

Figure S18. The raw data for electrocatalytic CO_2RR results. (a) GC-TCD, (b) GC-FID and (c) ^{1}H NMR spectra after applied potential at −0.9 V vs. RHE for 30 min in 0.1 M $KHCO_3$ aq. under CO_2 flow on Cu_{58}-I NC loaded catalysts. In (c), Water and DMSO were observed from an electrolyte and internal standard, respectively. For NMR analysis we used electrolyte 0.1 mL, D_2O 0.25 mL and DMSO 0.01 μL.

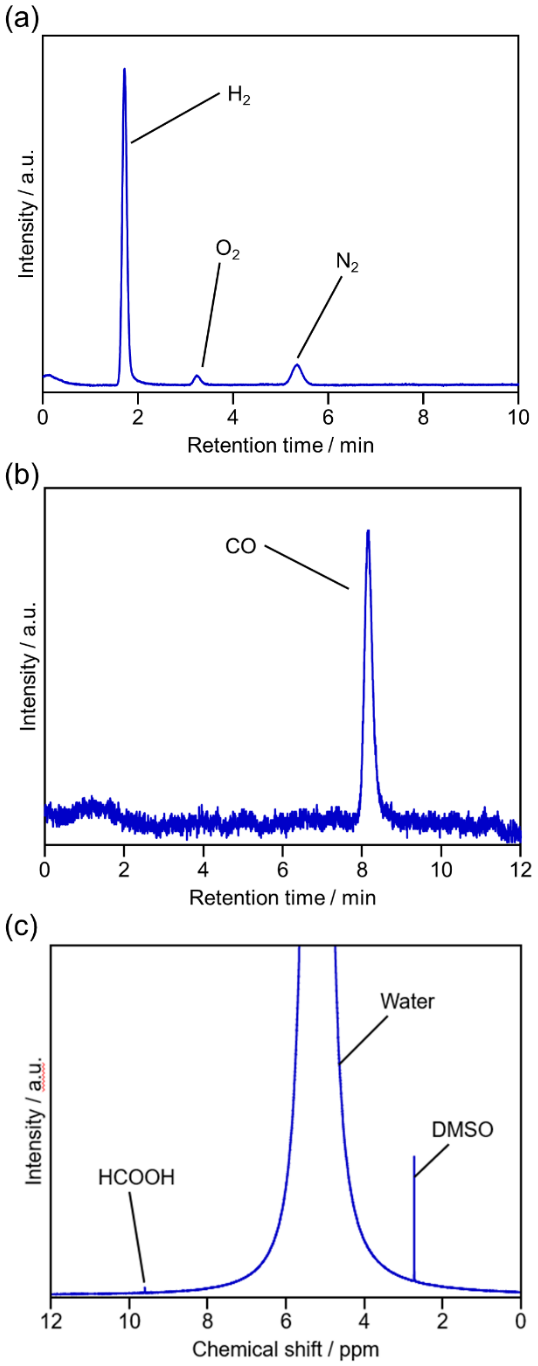

Figure S19. The raw data for electrocatalytic CO_2RR results. (a) GC-TCD, (b) GC-FID and (c) ^{1}H NMR spectra after applied potential at −0.9 V vs. RHE for 30 min in 0.1 M $KHCO_3$ aq. under CO_2 flow on Cu_{58}-II NC loaded catalysts. In (c), Water and DMSO were observed from an electrolyte and internal standard, respectively. For NMR analysis we used electrolyte 0.5 mL, D_2O 0.0995 mL and DMSO 0.05 μL.

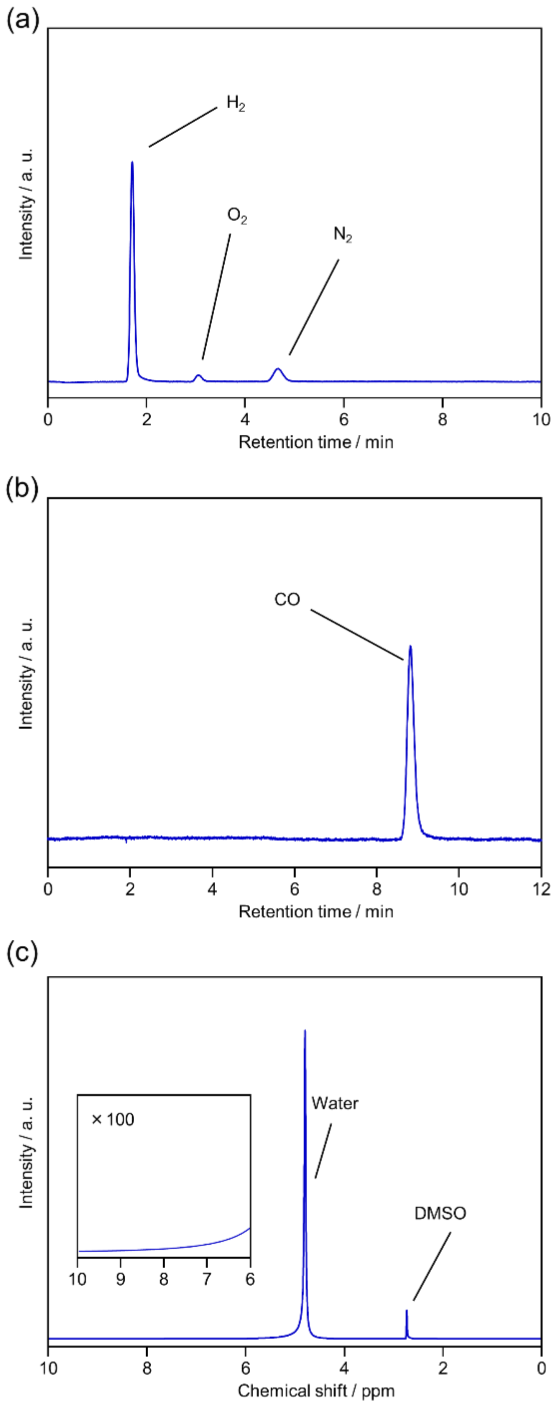

Figure S20. The raw data for electrocatalytic CO_2RR results. (a) GC-TCD, (b) GC-FID and (c) ^{1}H NMR spectra after applied potential at −0.9 V vs. RHE for 30 min in 0.1 M $KHCO_3$ aq. under CO_2 flow on Cu_{58} NC loaded catalysts. In (c), Water and DMSO were observed from an electrolyte and internal standard, respectively. For NMR analysis we used electrolyte 0.1 mL, D_2O 0.25 mL and DMSO 0.01 μL.

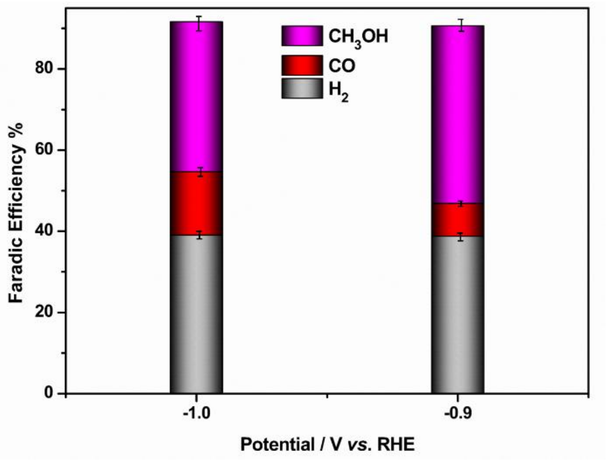

Figure S21. FE for CO_2 reduction products for Cu_{58}-I NC-loaded catalysts at different

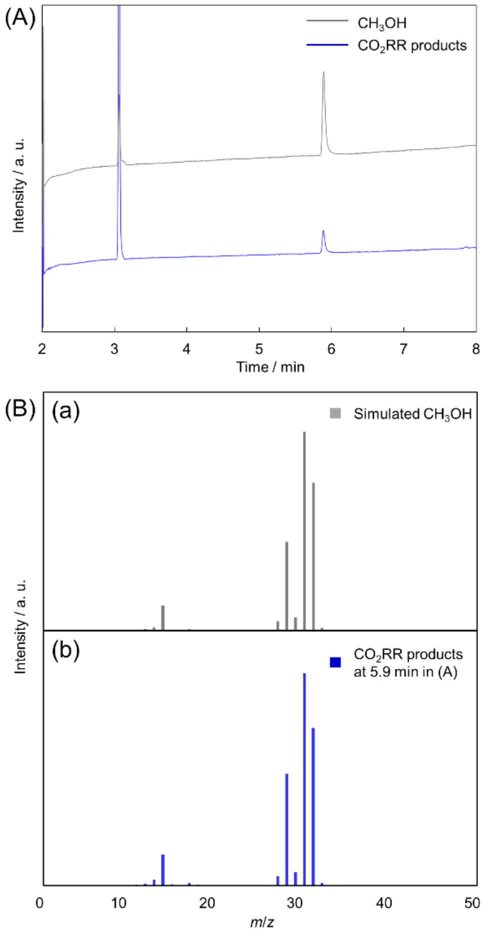

applied potentials.

Figure S22. Results of GC-MS analysis of electrocatalytic CO_2RR products in electrolyte for Cu_{58}-I NC-loaded catalysts applied potential at −0.9 V vs. RHE for 30 min in 0.1 M $KHCO_3$ aq. under CO_2 flow. (A) Chromatograms of CO_2RR products and CH_3OH as a reference and (B) mass spectrum of (a) the simulated ionization pattern of CH_3OH and (b) the peak at 5.9

min from the chromatograms in (A) of CO_2RR products. In (A), the strong peaks at 3.1 min is attributed from Ar gas for carrier.

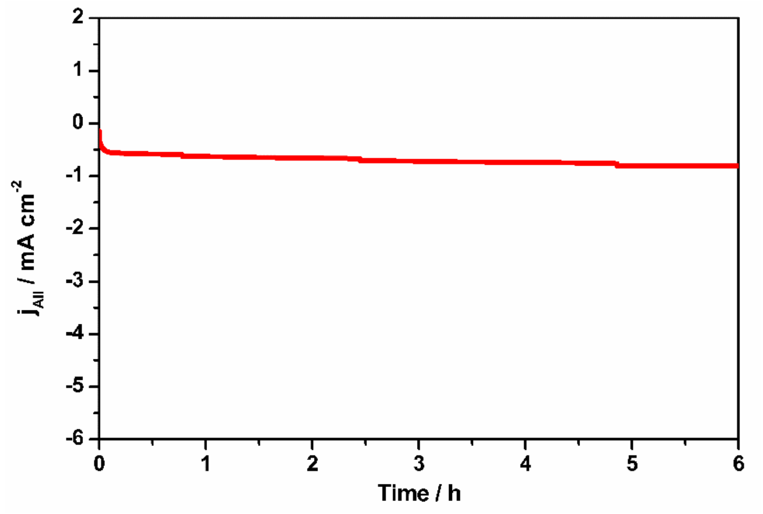

Figure S23. Stability tests of the Cu_{58}-I NC-loaded catalysts for CO_2RR at −0.9 V vs. RHE.

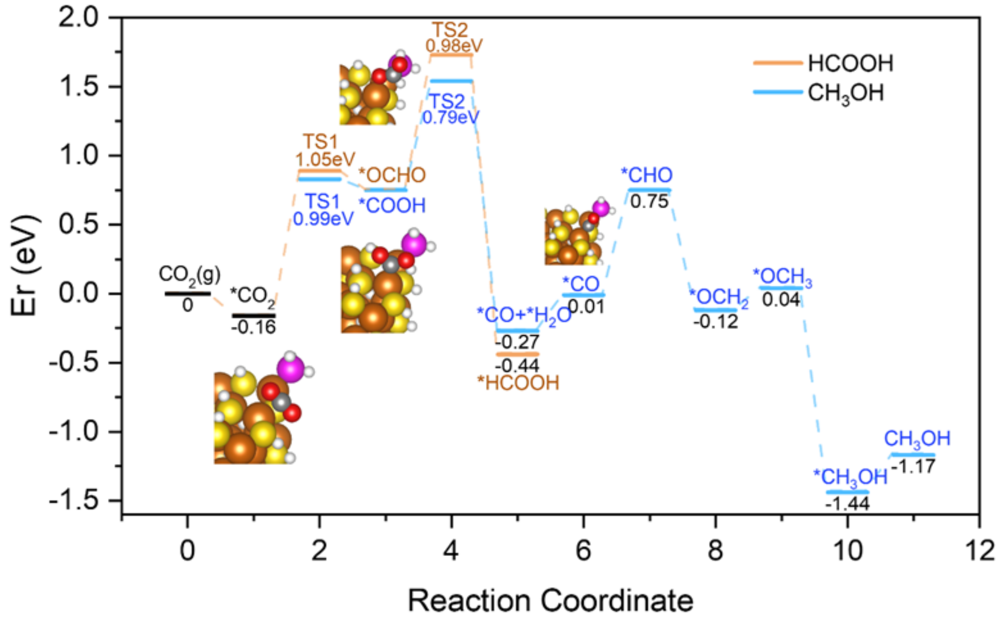

Figure S24. DFT-calculated energy profiles for CO_2 reduction reactions on Cu_{58}-I NC, leading to two different products (HCOOH and CH_3OH).

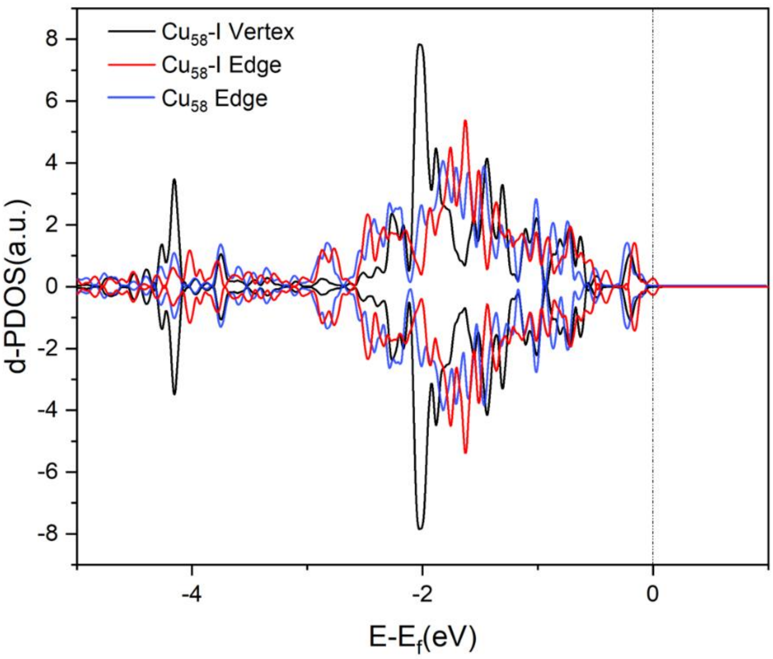

Figure S25. Projected density of states for the *d*-states of the different Cu sites on the Cu_{58}-I and Cu_{58} NCs.

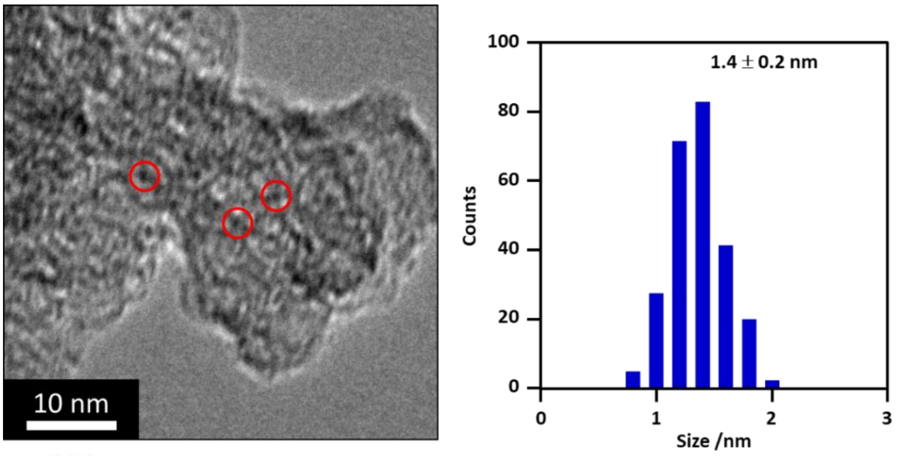

Figure S26. The TEM image and resulting size histograms of Cu_{58}-I NC on Cu_{58}-I NC/CB (a) before and (b) after 6 hours of electrocatalytic CO_2RR measurements.

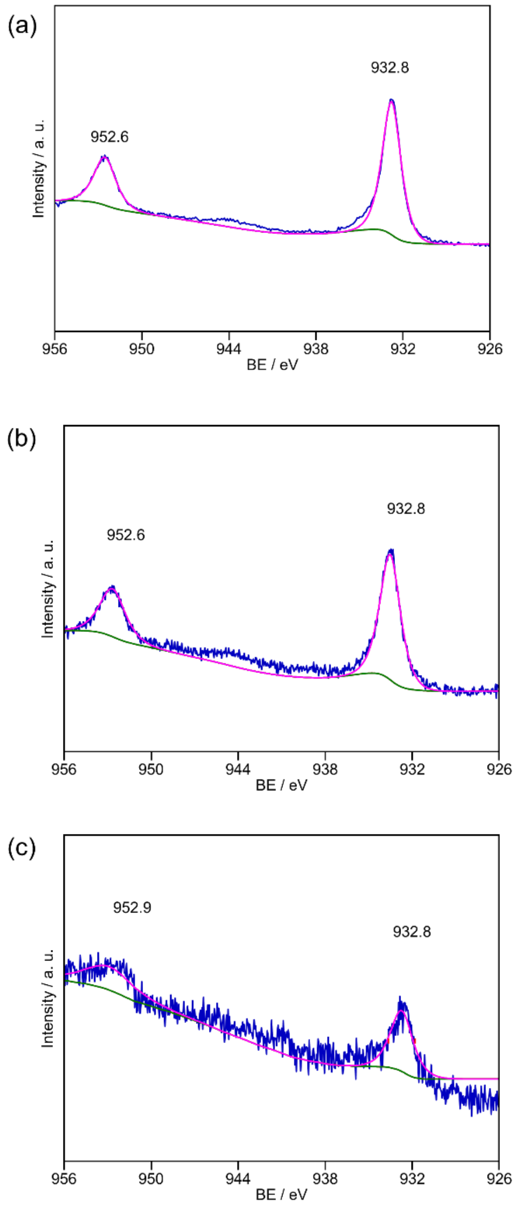

Figure S27. The Cu $2p_{1/2}$ (~953 eV) and $2p_{3/2}$ (~933 eV) XPS spectra of (a) Cu_{58}-I NC and Cu_{58}-I NC/CB (b) before and (c) after 6 hours of electrocatalytic CO_2RR measurements.

References

S1. H. Asakura, S. Yamazoe, T. Misumi, A. Fujita, T. Tsukuda, T. Tanaka, *Radiation Physics and Chemistry* **2020**, *175*, 108270.

S2. S. Biswas, S. Hossian, T. Kosaka, J. Sakai, D. Arima, Y. Niihori, M. Mitsui, D.-e. Jiang, S. Das, S. Wang, Y. Negishi, *Chem. Commun.* **2023**, *59*, 9336-9339.

S3. G. Kresse, J. Furthmüller, *Comput. Mater. Sci.* **1996**, *6*, 15-50.

S4. G. Kresse, J. Furthmüller, *Phys. Rev. B* **1996**, *54*, 11169.

S5. J. P. Perdew, K. Burke, M. Ernzerhof, *Phys. Rev. Lett.* **1996**, *77*, 3865.

S6. S. Grimme, J. Antony, S. Ehrlich, H. Krieg, *J. Chem. Phys.* **2010**, *132*.

S7. G. M. Sheldrick, *Acta Crystallogr., Sect. C: Struct. Chem.* **2015**, *71*, 3-8.

S8. Bruker APEX3, v2019.1–0, Bruker AXS Inc., Madison, WI, USA, (2019).

S9. O. V. Dolomanov, L. J. Bourhis, R. J. Gildea, J. A. Howard, H. Puschmann, *J. Appl. Crystallogr.* **2009**, *42*, 339-341.
